# A BMP-controlled metabolic/epigenetic signaling cascade directs midfacial morphogenesis

**DOI:** 10.1172/JCI165787

**Published:** 2024-03-11

**Authors:** Jingwen Yang, Lingxin Zhu, Haichun Pan, Hiroki Ueharu, Masako Toda, Qian Yang, Shawn A. Hallett, Lorin E. Olson, Yuji Mishina

**Affiliations:** 1 State Key Laboratory of Oral & Maxillofacial Reconstruction and Regeneration, Key Laboratory of Oral Biomedicine Ministry of Education, Hubei Key Laboratory of Stomatology, School and Hospital of Stomatology, Wuhan University, Wuhan, Hubei, China.; 2 Department of Biologic and Materials Sciences, School of Dentistry, and; 3 Life Sciences Institute, University of Michigan, Ann Arbor, Michigan, USA.; 4 Cardiovascular Biology Research Program, Oklahoma Medical Research Foundation, Oklahoma City, Oklahoma, USA.

**Keywords:** Development, Therapeutics, Embryonic development, Mouse models

## Abstract

Craniofacial anomalies, especially midline facial defects, are among the most common birth defects in patients and are associated with increased mortality or require lifelong treatment. During mammalian embryogenesis, specific instructions arising at genetic, signaling, and metabolic levels are important for stem cell behaviors and fate determination, but how these functionally relevant mechanisms are coordinated to regulate craniofacial morphogenesis remain unknown. Here, we report that bone morphogenetic protein (BMP) signaling in cranial neural crest cells (CNCCs) is critical for glycolytic lactate production and subsequent epigenetic histone lactylation, thereby dictating craniofacial morphogenesis. Elevated BMP signaling in CNCCs through constitutively activated ACVR1 (ca-ACVR1) suppressed glycolytic activity and blocked lactate production via a p53-dependent process that resulted in severe midline facial defects. By modulating epigenetic remodeling, BMP signaling–dependent lactate generation drove histone lactylation levels to alter essential genes of *Pdgfra*, thus regulating CNCC behavior in vitro as well as in vivo. These findings define an axis wherein BMP signaling controls a metabolic/epigenetic cascade to direct craniofacial morphogenesis, thus providing a conceptual framework for understanding the interaction between genetic and metabolic cues operative during embryonic development. These findings indicate potential preventive strategies of congenital craniofacial birth defects via modulating metabolic-driven histone lactylation.

## Abstract 2

A BMP-controlled metabolic-epigenetic signaling cascade directs midfacial morphogenesis in mice.

## Graphical abstract

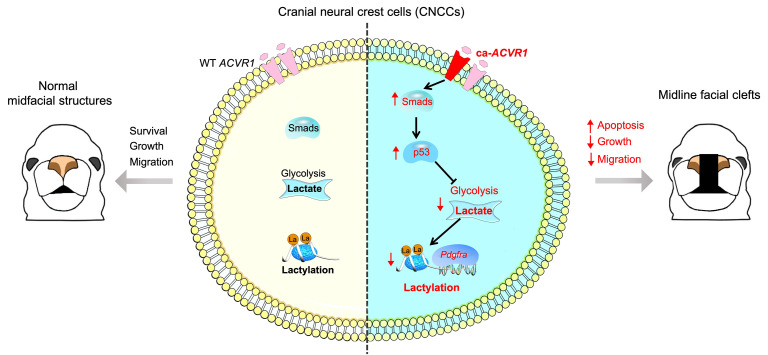

## Introduction

Craniofacial anomalies, especially midline facial defects, are among the most common birth defects found in patient populations whose appearance are either associated with increased mortality or require lifelong treatment (1–3). The majority of craniofacial tissues are derived from cranial neural crest cells (CNCCs), which arise at the dorsal tip of the closing neural tube and migrate along stereotypical paths to populate craniofacial primordia (3, 4). Craniofacial morphogenesis governed by CNCCs involves a very complex series of events that requires a very high degree of coordination and precise timing of cellular delamination, migration, growth, and stem cell differentiation (3). Despite advances in human genome sequencing technologies, the causes of nearly 70% of all birth defects, including midline facial defects, remain unknown (3).

Histone posttranslational modifications (HTMs) are crucial epigenetic mechanisms regulating gene expression in various biological events. Cellular chromatin is composed of DNA and histones. Histones can undergo a wide range of HTMs, such as phosphorylation, methylation, acetylation, and other acylation modifications. HTMs regulate expression of genes involved in inflammation, cancer, embryonic development, cardiovascular diseases, kidney diseases, metabolic diseases, and neuropsychiatric disorders (5–7). Lactate-derived lactylation of histone lysine residues (histone lactylation) is a recently identified posttranslational modification that directly stimulates gene expression (8). Notably, histone lactylation has been reported to play a vital role in cancer, inflammation, and stem cell pluripotency (9–12). However, the potential function of histone lactylation in embryonic development remains unknown.

Bone morphogenetic protein (BMP) signaling is highly conserved across many species and is important in regulating embryogenesis, skeletal development, and the maintenance of adult-tissue homeostasis (13–16). However, the potential contribution of BMP signaling to midline facial morphogenesis remains undefined. Interestingly, recent studies have demonstrated that shifts in carbon metabolism play essential roles in regulating cellular function during embryogenesis (17, 18). New evidence suggests that the intermediate metabolites, including acetyl–coenzyme A (acetyl-CoA), α-ketoglutarate (α-KG), and lactate, can directly determine cell fate and function via epigenetically modifying histone acetylation, histone and DNA demethylation, or histone lactylation (8, 19, 20). The possibility that BMP signaling functionally integrates genetic and metabolic cues to affect CNCC development — in vitro or in vivo — remains unexplored. Herein, we demonstrate that BMP signaling unexpectedly contributes to midline facial morphogenesis by modulating a p53-dependent control of glycolytic lactate production that in turn regulates histone lactylation levels to alter essential genes of *Pdgfra*. Our findings here define an axis wherein BMP signaling controls a metabolic/epigenetic transcascade to direct craniofacial morphogenesis, thus providing a conceptual framework for understanding the interaction between genetic and metabolic cues operative during embryonic development. Importantly, manipulation of this metabolic-driven histone lactylation prevents congenital craniofacial deformities and offers what we believe to be a unique opportunity for the prevention of craniofacial malformations.

## Results

### Elevated BMP/Smad signaling in CNCCs elicits drastic midline facial defects.

**Figure 1:**
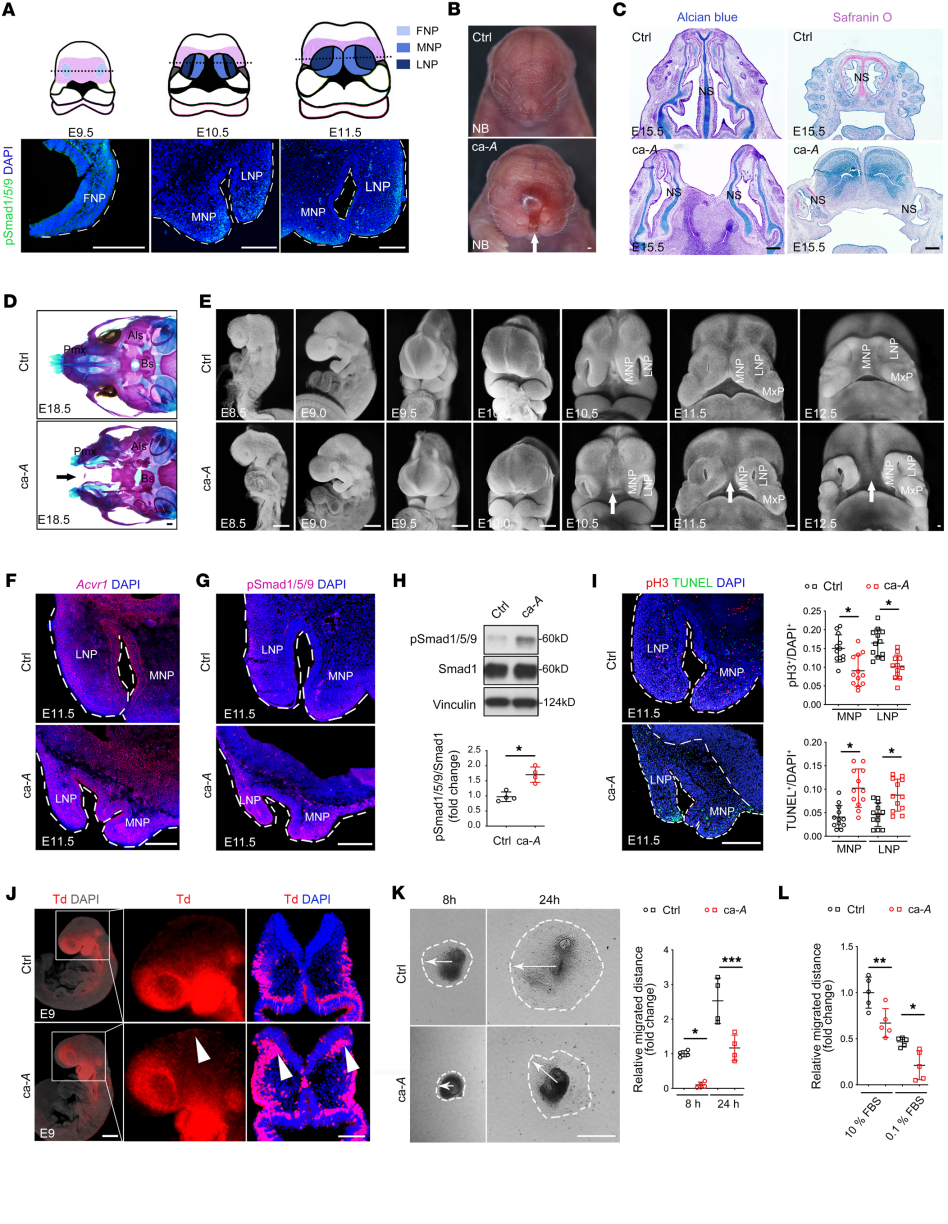
Elevated BMP/Smad signaling causes midline facial defects via regulating CNCC growth and migration in a stage-dependent manner. (**A**) Drawing showing the morphogenesis of midfacial structures (upper) and pSmad1/5/9 immunostaining in NP tissues (lower, *n* = 4). Black dotted lines in the upper panel indicate approximate position of each section in the lower panel. (**B**) Frontal view of facial structures in control and ca-*Acvr1(A11)* (abbreviated as ca-*A* in figures) mutants at NB stage (*n* = 11). (**C**) Alcian blue–stained transverse sections and safranin O–stained frontal sections of facial structures (*n* = 4). (**D**) Ventral view of whole-mount alizarin red– and Alcian blue–stained heads (*n* = 9). (**E**) Lateral and frontal view of DAPI-stained heads (*n* = 5). (**F** and **G**) *Acvr1* RNA Scope (**F**) and pSmad1/5/9 immunostaining (**G**) of NP tissues (*n* = 5). (**H**) Representative immunoblots and quantification results of pSmad1/5/9 and Smad1 in E11.5 NP tissues (*n* = 4). (**I**) pH3 immunofluorescence (red), TUNEL staining (green), and quantification results in NP tissues (*n* ≥ 4). (**J**) Lateral view (left and middle) and coronal sections (right) of *R26R^tdTomato^*-positive embryos at E9 (*n* =4). White arrowheads indicate dorsal end locations of CNCCs. (**K**) Explant cultures and quantification of CNCCs emigrating from E8.5 neural crest explants (*n* = 4). (**L**) Wound-scratch assay and quantification of cell migration distance of NP cells (*n* = 5). For all panels, data are represented as means ± SD. *P < 0.05; **P < 0.01; ***P < 0.001, unpaired 2-tailed Student’s *t* test (**H**, **I**, **K**, and **L**). Scale bars: 200 μm. NS, nasal septum; Pmx, premaxilla; Bs, basisphenoid; Als, alisphenoid; MxP, maxillary process. White and black arrows indicate midline facial cleft (**B**, **D**, and **E**).

Midfacial morphogenesis depends upon the growth and fusion of several tissue rudiments, including the frontonasal prominence (FNP) and both the paired lateral and medial nasal processes (LNP and MNP, respectively), with merging of the MNP processes giving rise to midline facial structures (3, 21) (Figure 1A). As *Noggin* loss-of-function mutations, which potentially lead to enhanced BMP signaling, induce bifid nasal pits in humans (22), we first examined the dynamic activation and distribution of pSmad1/5/9 in the FNP, MNP, and LNP regions during normal midfacial development. pSmad1/5/9 was barely detected in the FNP at E9.5, but at E10.5, was preferentially localized to the distal tips of MNP and LNP mesenchyme rather than the ventral section of the MNP (Figure 1A). Then, at E11.5, pSmad1/5/9 was mainly distributed in the lateral regions of the LNP mesenchyme, where it seemed weaker than that at E10.5 (Figure 1A). These results suggest that BMP/Smad signaling is precisely regulated in a temporospatial manner during development of the midfacial structures.

Previous lineage studies have revealed that migrating CNCCs make up the majority of mesenchymal cell populations found within MNP and LNP (21, 23). While the expression of constitutively activated *Bmpr1a* in CNCCs did not affect overt midfacial structures (24–26), we sought to define the in vivo impact of increased BMP/Smad signaling via ACVR1, another type I BMP receptor, on midfacial morphogenesis. To this end, we generated a transgenic mouse model with a floxed constitutively activated *Acvr1* allele (line A11, ca-*Acvr1(A11)*) (27) and crossed it with *P0-Cre* (28) to enhance BMP/Smad signaling in a neural crest–specific manner. Unexpectedly, we found that over 85% of the ca-*Acvr1(A11)* mutants (ca-*Acvr1(A11)^fl/+^;P0-Cre*) displayed drastic midline facial defects, including a midline facial cleft, missing primary palate, bifurcated nasal septum, and short snout (Figure 1, B and C, and Supplemental Figure 1, A–C; supplemental material available online with this article; https://doi.org/10.1172/JCI165787DS1). At midgestation, ca-*Acvr1(A11)* mutants lacked the philtrum and the primary palate (Figure 1C and Supplemental Figure 1B), both of which are derived from the MNP (3). At E18.5, whole-mount skeletal staining showed that ca-*Acvr1(A11)* mutant embryos exhibited malformation of the midline facial clefts and neural crest–derived structures, such as premaxilla, basisphenoid, and alisphenoid (Figure 1D). None of the ca-*Acvr1(A11)* mutants with facial clefts survived beyond perinatal stages. We were also able to recapitulate the midline facial defects phenotype in mice carrying another neural crest–specific Cre driver, *Wnt1-Cre* (ca-*Acvr1[A11]^fl/+^;Wnt1-Cre*; Supplemental Figure 1D). Together with the fact that transgenic mice with increased BMP/Smad signaling in the epithelium (ca-*Acvr1; K14-Cre*) showed a normal midline facial structure (29), our results suggest that BMP/Smad signaling in the mesenchyme, but not the epithelium, is critical for the midline facial cleft phenotype.

In monitoring the morphological differences observed at early stages when MNP and LNP are formed (3), we noted that morphological differences first became visible at E10.5 when the MNP and LNP in ca-*Acvr1(A11)* mutants were smaller and the gap between processes was larger in mutant embryos compared with controls (Figure 1E), while the gross craniofacial morphology was comparable between control and mutants at E8.5, E9.0, E9.5, and E10.0 (Figure 1E). Phenotypic differences became more obvious at E11.5 and E12.5, as the MNP merged in control embryos but remained separated in ca-*Acvr1(A11)* mutants (Figure 1E). As expected, *Acvr1* mRNA expression was increased in the NPs of ca-*Acvr1(A11)* mutants (Figure 1F) with elevated pSmad1/5/9 levels (Figure 1, G and H, and Supplemental Figure 1E). However, noncanonical BMP signaling pathways, including pERK, pJNK, pP38 MAPK, and pTAK1, were unaffected in the nasal process (NP) tissues of ca*-Acvr1(A11)* mutants (Supplemental Figure 1F). Suppressing BMP/Smad signaling using a suboptimal dose of LDN193189, a specific inhibitor of the type I BMP receptor (30), alleviated the facial clefts observed in ca-*Acvr1(A11)* mutants (Supplemental Figure 1, G and H). Of note, a single injection of LDN193189 at E10.75 and E11.25 rescued more ca-*Acvr1(A11)* mutants than when injected at other stages (Supplemental Figure 1I), suggesting E10.5–E11.5 are the most vulnerable stages for the midline facial cleft phenotype.

Next, we analyzed cell proliferation and apoptosis in developing CNCCs as well as their derivatives in NP tissues. Strikingly, cell proliferation was significantly decreased, as assessed by pH3 staining, while the number of TUNEL-positive apoptotic cells was increased in mesenchymal cells of mutant MNP and LNP compared with controls at E11.5 (Figure 1I). In contrast, epithelial cell proliferation and apoptosis were not affected in mutants (analysis data not shown). A similar trend was found for cell proliferation and apoptosis in the mesenchyme of mutant FNP tissues at E9.5 (Supplemental Figure 2A), but CNCC proliferation and apoptosis were unaltered in mutants at E8.5 and E9.0 (Supplemental Figure 2B). BMP signaling plays important roles in chondrogenic, osteogenic, and neurogenic differentiation of stem cells (31). However, the distribution of the osteogenic marker Osx, the chondrogenic marker Sox9, and the sensory neuronal marker Neurofilament did not show overt changes in ca-*Acvr1(A11)* NP tissues at E11.5 (Supplemental Figure 2C), suggesting that CNCC differentiation was not significantly altered in ca-*Acvr1(A11)* mutants.

We further traced the migration of CNCCs by introducing the *R26R^Td-Tomato^* reporter allele (32) in both control and ca-*Acvr1(A11)* mutants. Both *R26R^Td-Tomato^* reporter signal distribution and neural crest–marker genes (*Sox10* and *Ap2*α) were comparable in mutant and control embryos at E8.5 (Supplemental Figure 2, D and E), indicating normal neural crest formation. Although CNCC migration to destination sites was comparable between mutants and controls at E11.5 and E9.5 (Supplemental Figure 2D), more *R26R^Td-Tomato^*–positive cells were located in the dorsal portion of mutant heads than in controls at E9.0 (Figure 1J), suggesting the retarded migration of mutant CNCCs. To assess migration defects ex vivo, explant cultures were established from the cranial neural tubes of mutant and control embryos at E8.5 in the presence of mitomycin C to block proliferative responses (33). The mutant explant cultures exhibited a significantly decreased migratory response for mutant CNCCs relative to controls (Figure 1K). Similarly, we found that the migration distance was significantly reduced in primary CNCCs isolated from the NP tissues (NP cells) from ca-*Acvr1(A11)* embryos compared with controls (Figure 1L). Together, these results indicate that elevated BMP/Smad signaling via constitutively activated ACVR1 in CNCCs causes drastic midline facial defects with reduced growth and retarded CNCC migration.

### Enhanced BMP/Smad signaling suppresses glycolytic lactate production and histone lactylation in CNCCs during midline facial development.

**Figure 2:**
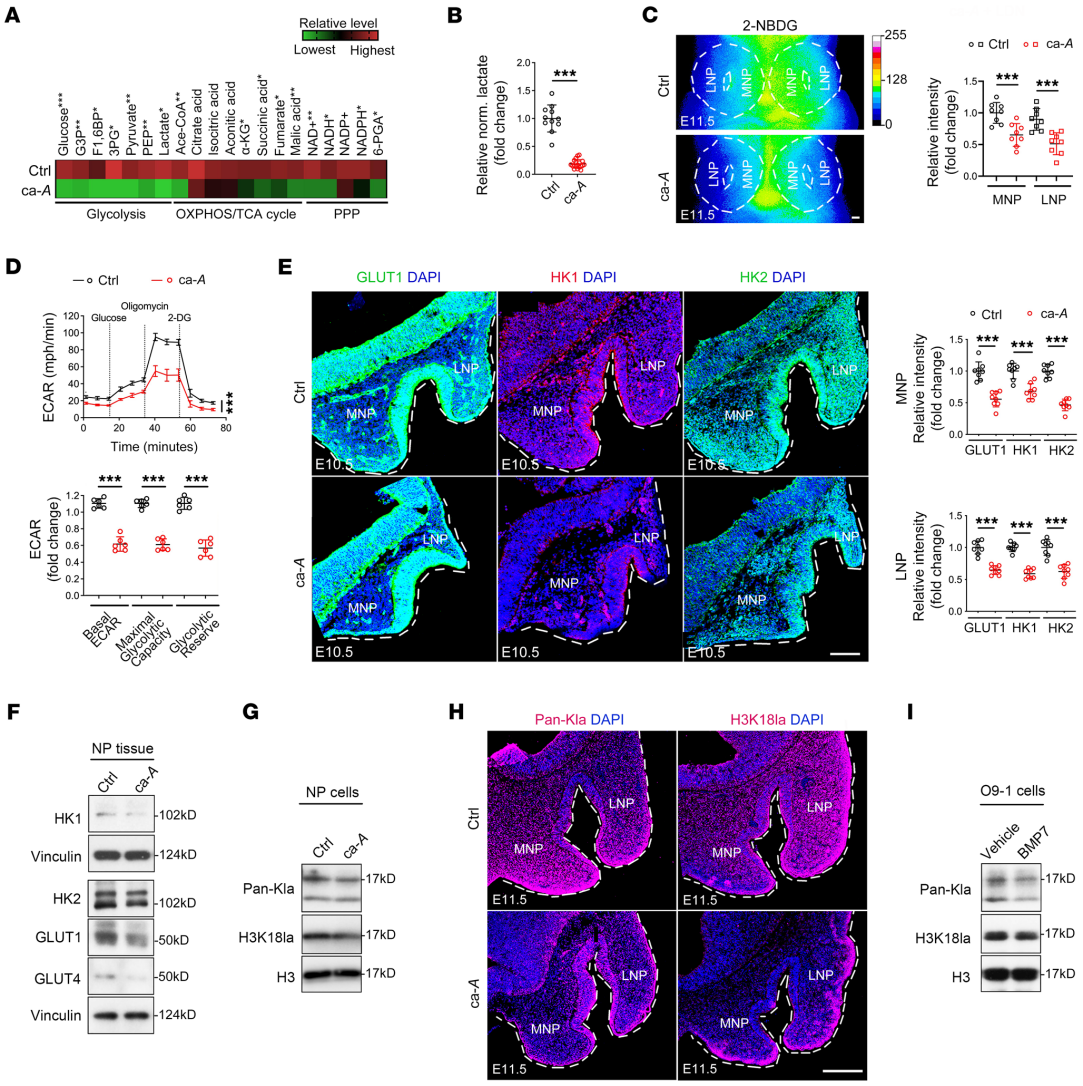
Enhanced BMP/Smad signaling suppresses glycolytic activity and reduces lactate-derived histone lactylation in CNCCs during midline facial development. (**A**) Expression profiles of the metabolites associated with glucose metabolism in NP cells (*n* = 3). The color bar shows the fold change from the mean of all triplicate samples. (**B**) Relative normalized (norm.) lactate levels in E11.5 NP tissues (*n* ≥ 11). (**C**) Frontal view of pseudocolor images (16 colors) using ImageJ (NIH) and intensity quantification of fluorescent glucose (2-NDBG) in E11.5 NP tissues (*n* = 8). (**D**) ECAR measurements and calculated glycolytic flux and glycolytic capacity in NP cells (*n* = 6). (**E**) Representative immunostaining images and intensity quantification of GLUT1 (green, left), HK1 (red, middle), and HK2 (green, right) in E10.5 NP tissues (*n* = 4). (**F**) Representative immunoblots of GLUT1, GLUT4, HK1, and HK2 in E11.5 NP tissues (*n* = 4). Results shown are from blots run contemporaneously. (**G**) Representative immunoblots of Pan-Kla and H3K18la in E11.5 NP tissues (*n* = 5). Results shown are from blots run contemporaneously. (**H**) Pan-Kla (red, left) and H3K18la (red, right) immunostaining in E11.5 NP tissues (*n* = 4). (**I**) Representative immunoblots of Pan-Kla and H3K18la in O9-1 cells treated with or without BMP7 (*n* = 5). Results shown are from blots run contemporaneously. For all panels, data are represented as means ± SD. ***P < 0.001, unpaired 2-tailed Student’s *t* test (**B**–**E**). Scale bars: 100 μm (**C**, **E**, and **H**). G3P, glucose 3-phosphate; F1,6BP, fructose 1,6-bisphosphate; 3BP, 3-phosphoglyceric acid; PEP, phosphoenolpyruvic acid; Ace-CoA, acetyl-CoA.

To explore downstream mechanisms of how BMP/Smad signaling is involved in midfacial development, we next set up bulk RNA-Seq replicates on NP tissues from E11.5 control and ca-*Acvr1(A11)* mutants and found 131 upregulated genes and 410 downregulated genes in ca-*Acvr1(A11)* mutants. The initial Kyoto Encyclopedia of Genes and Genomes (KEGG) enrichment analysis results showed that metabolic pathways are the second top signaling pathways affected in ca-*Acvr1(A11)* mutants following the PI3K/AKT signaling pathway. The other gene groups affected are focal adhesion/cytoskeleton and the MAPK signaling pathway following metabolic pathways (Supplemental Figure 3A). Among them, we focused on metabolic pathways because during development, embryonic cells shift their metabolism to meet the energy and signaling requirements necessary to support cell growth and motility (17, 34, 35). Quantitative reverse-transcription PCR (qRT-PCR) data confirmed that the majority of genes related to glycolysis are significantly downregulated in mutant NP tissues at E10.5 (from glucose transporters to enzymes to generate pyruvate; Supplemental Figure 3B). Because of a complementary body of literature linking BMP signaling to metabolic control in other cell systems (16, 36–38), we performed metabolomic analyses of carbon metabolism, including glycolysis, TCA cycle, and pentose phosphate pathway (PPP), in control and ca-*Acvr1(A11)* NP cells by liquid chromatography–mass spectrometry (LC-MS). Notably, metabolites of the glycolytic pathway (i.e., pyruvate, lactate, etc.) were among the most significantly decreased in ca-*Acvr1(A11)* mutants (Figure 2A), while metabolites of the TCA cycle (i.e., acetyl-CoA, citrate, isocitrate, etc.) and the PPP (6-phosphogluconic acid [6-PGA], NAD^+^, NADP^+^, etc.) were only modestly decreased (Figure 2A). Therefore, we focused on the glycolytic pathway in this study. By monitoring glucose uptake and lactate levels dynamically, we confirmed that glucose uptake and lactate levels were decreased in ca-*Acvr1(A11)* mutant NP cells and NP tissues, respectively (Supplemental Figure 3C and Figure 2B), findings consistent with the decreased uptake of the fluorescent glucose analog 2-NBDG in ca-*Acvr1(A11)* NP tissues (Figure 2C). Further, primary NP cells from ca-*Acvr1(A11)* mutants displayed a significantly lower extracellular acidification rate (ECAR) and glycolytic reserve (the difference between the maximum glycolytic capacity and the basal glycolytic rate) than control NP cells (Figure 2D), suggesting reduced glycolytic activity in mutant cells. In contrast, the oxygen consumption rate (OCR), mitochondrial DNA copy number, mitochondria amount, and levels of complexes involved in oxidative phosphorylation (OXPHOS) were unaffected in ca-*Acvr1(A11)* mutants (Supplemental Figure 3, D–G). Consistent with the metabolic data and decreased gene expression, ca-*Acvr1(A11)* mutant NP tissues exhibited remarkably decreased protein levels of key mediators of glycolysis, such as solute carrier family 2 member 1 (Slc2a1), Slc2a4, hexokinase-1 (Hk1), and Hk2 (Figure 2, E and F). Of note, decreases in protein levels and gene expression of key mediators of glycolysis were detected as early as E9 (Supplemental Figure 3, H and I), a time point when cell proliferation and apoptosis were not yet altered in ca-*Acvr1(A11)* mutant embryos (Supplemental Figure 2B). To substantiate the role of BMP signaling in regulating histone lactylation, we stimulated the O9-1 CNCC cell line (39) with BMP7. The results showed that enhanced BMP signaling via BMP7 treatment suppressed glycolytic gene expression and the ECAR level, while the OCR level and protein levels of the main OXPHOS complexes were unaltered (Supplemental Figure 3, J–M).

In addition to providing fuel for metabolic activity, glycolysis leads to the production of metabolites that can modify transcriptional responses (8, 10, 19, 20). Acetyl-CoA and α-KG have been found to regulate the epigenome via histone acetylation and demethylation, respectively, thereby affecting cell-fate determination (19, 20). Recently, lactate-mediated histone lactylation has been reported as a new epigenetic modification linking glucose metabolism to gene expression (8). Therefore, the decreases observed in CNCC acetyl-CoA, α-KG, and lactate levels after BMP activation (Figure 2A) prompted us to test to determine whether BMP signaling affects epigenetic changes during CNCC growth/migration and midline facial development. In this regard, the protein levels of histone lactylation markers, including lactylated lysine/K (Pan-Kla) and H3K18la, were decreased significantly in ca-*Acvr1(A11)* NP tissues at E11.5 (Figure 2G), while histone methylation markers (H3K9me3 and H3K27me3) and acetylation markers (H3K9Ac, H3K14Ac, H3K18Ac, H3K27Ac, and Pan-Kac) were unaltered (Supplemental Figure 4A). The reductions in both Pan-Kla and H3K18la in mesenchymal cells of ca-*Acvr1(A11)* NP tissues were confirmed by immunostaining (Figure 2H). As predicted, BMP7 treatment significantly decreased the protein abundance of Pan-Kla and H3K18la in O9-1 cells (Figure 2I). In vivo administration of LDN193189 potentiated the expression of glycolytic mediators, lactate levels, Pan-Kla, and H3K18la levels in NP tissues of ca-*Acvr1(A11)* mutants (Supplemental Figure 4, B–D). Hence, enhanced BMP/Smad signaling negatively regulates glycolytic lactate production and histone lactylation in CNCCs during midfacial development.

### Recovery of glycolytic activity or lactate levels rescues histone lactylation and facial defects of ca-ACVR1 mutants.

**Figure 3:**
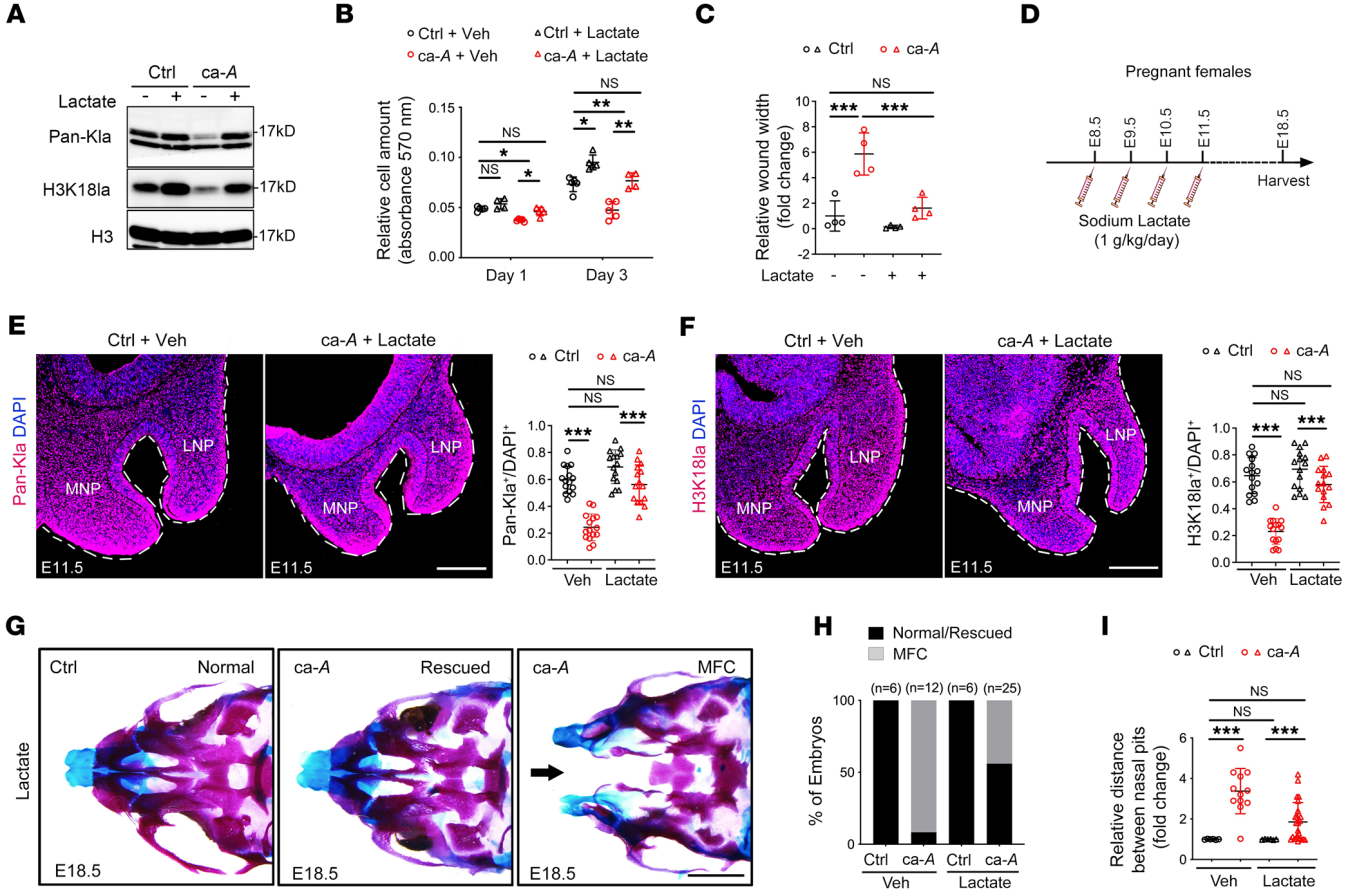
Reduced lactate production and histone lactylation in CNCCs is responsible for the midline facial defects of ca-*Acvr1(A11)* mutants. (**A**) Representative immunoblots of Pan-Kla and H3K18la in control and ca-*Acvr1(A11)* NP cells treated with or without sodium lactate (*n* = 3). Results shown are from blots run contemporaneously. (**B** and **C**) Quantification of cell-proliferation assay (**B**) and wound-scratch assay (**C**) of control and ca-*Acvr1(A11)* NP cells treated with or without sodium lactate (*n* ≥ 4). (**D**) Schematic diagram showing the i.p. injection schedule of sodium lactate (1 g/kg/d, E8.5–E11.5) into pregnant females. (**E** and **F**) Pan-Kla (red, **E**) and H3K18la (red, **F**) immunofluorescence and quantification of positive cell percentage in NP tissues of embryos treated with or without sodium lactate (1 g/kg/d, E8.5–E11.5, *n* = 6). (**G**–**I**) Whole-mount skeletal staining (**G**) and quantification of the embryo number (**H**) and distance between nasal pits (**I**) of control and ca-*Acvr1(A11)* embryos treated with or without sodium lactate (1 g/kg/d, E8.5–E11.5, *n* ≥ 6). For all panels, data are represented as means ± SD. *P < 0.05; **P < 0.01; ***P < 0.001, 1-way ANOVA (**B**, **C**, **E**, **F**, and **I**). Scale bars: 100 μm (**E** and **F**); 1 mm (**G**). Black arrow indicates midline facial cleft (**G**).

We then sought to determine whether reduced lactate production and histone lactylation in CNCCs are responsible for midline facial defects of ca-*Acvr1(A11)* mutants. Given that exogenous sodium lactate has been reported as promoting histone lactylation in macrophages cultured in vitro (8), we examined the effect of lactate supplementation on histone lactylation and subsequently the defects of ca-*Acvr1(A11)* mutant cells. Lactate supplementation successfully rescued histone lactylation without affecting pSmad1/5/9 levels in ca-*Acvr1(A11)* NP cells in culture (Figure 3A and Supplemental Figure 4E), while in tandem fashion, restoring cell growth or migration to levels comparable to those of control NP cells (Figure 3, B and C). The function of lactate in CNCC growth and migration was also confirmed in O9-1 cells transfected with *Ldha* siRNA or treated with the LDHA-specific inhibitor GNE-140 (Supplemental Figure 4, F–H). In contrast, exogenous sodium acetate failed to ameliorate the growth or migration defects of ca-*Acvr1(A11)* NP cells (Supplemental Figure 4, I and J), although slightly decreased acetyl-CoA levels were noted in ca-*Acvr1(A11)* NP tissues (Figure 2A). To further determine whether a reduction in lactate-mediated histone lactylation is the critical mechanism for midline facial defects, we administered sodium lactate to pregnant females from E8.5 to E11.5 via i.p. injection (Figure 3D). Sodium lactate administration rescued the protein distribution of Pan-Kla and H3K18la (Figure 3, E and F) and the midline facial structures of the majority (>50%) of ca-*Acvr1(A11)* mutants (Figure 3, G and H). Sodium lactate treatment also significantly reduced the distance between nasal pits in ca-*Acvr1(A11)* mutants relative to vehicle treatment (Figure 3I). Notably, there were no obvious negative effects of sodium lactate injection on fertility and lactation activity of females based on their litter size and postnatal growth (Supplemental Figure 5, A and B). Pups whose facial abnormalities were rescued by exogenous sodium lactate can survive postnatally (no additional death between P1 and P42, total of >50% of ca-*Acvr1[A11]* mutants at NB [newborn stage], while only 10% of the mutants were found surviving at P1 and P42) (Supplemental Figure 5, C and D). Females that received lactate were bred again without additional treatment, and they showed comparable levels of litter size (Supplemental Figure 5A) and postnatal growth of the pups (Supplemental Figure 5B), suggesting that lactate treatment does not affect second pregnancy either. Given these results, we finally sought to determine whether overexpression of *Slc2a1* and/or *Hk2* in primary ca-*Acvr1(A11)* mutant NP cells might restore glycolytic activity and lactate production. Transfection of *Slc2a1* and *Hk2* plasmid significantly increased the protein amount of GLUT1 and HK2 in NP cells, respectively (Supplemental Figure 6A), while forced expression of either *Slc2a1* or *Hk2* in ca-*Acvr1(A11)* mutant NP cells restored lactate levels and significantly alleviated defects in cell growth and migration (Supplemental Figure 6, B–D). Taken together, these findings suggest that reduced lactate production and histone lactylation downstream of glycolytic activity in CNCCs are responsible for the midline facial defects observed in ca-*Acvr1(A11)* mutants.

### BMP-mediated regulation of Pdgfra histone lactylation directs CNCC and facial development.

**Figure 4:**
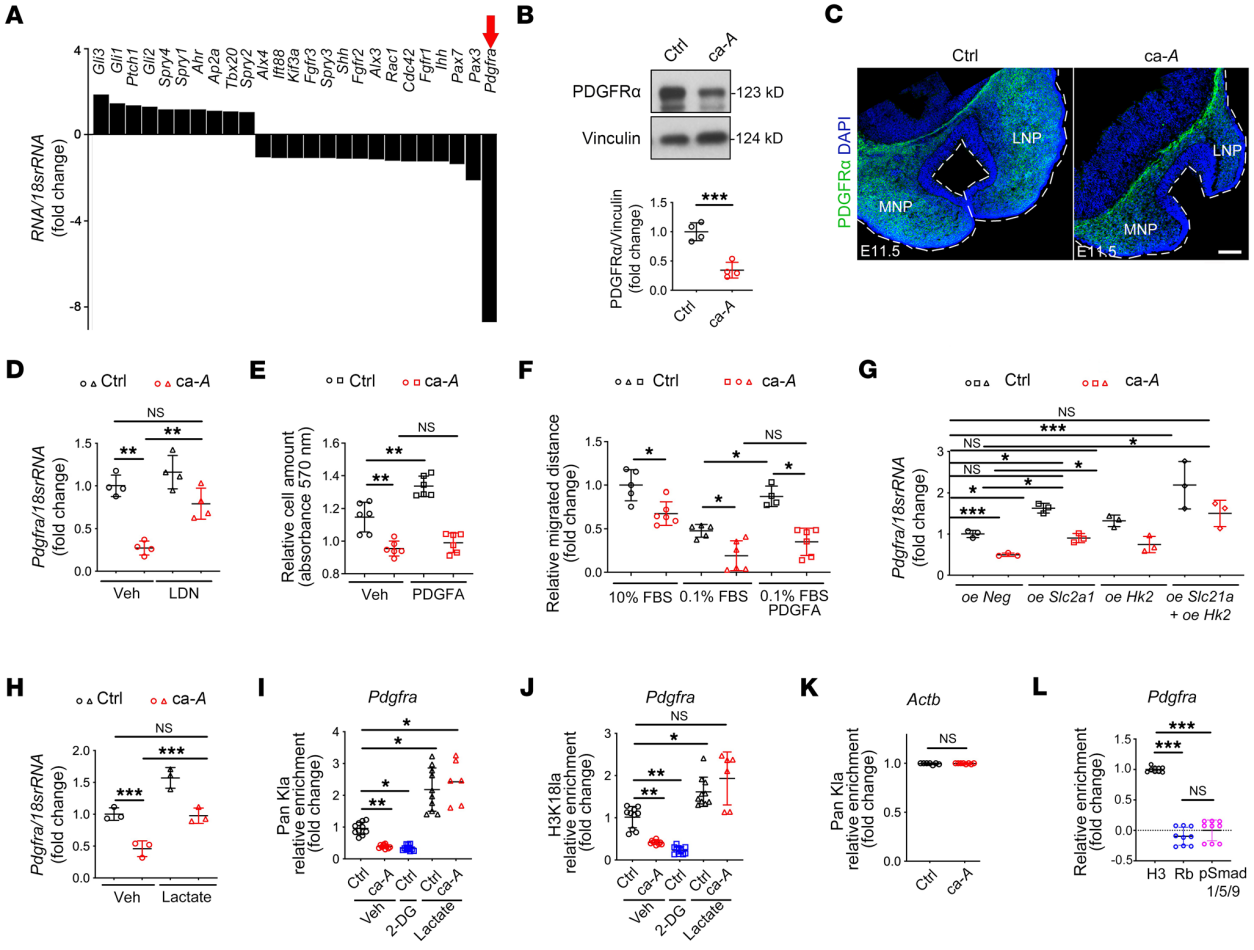
BMP-mediated regulation of histone lactylation in *Pdgfra* gene directs CNCC behaviors. (**A**) Relative mRNA expression of critical genes for midline facial development in E11.5 NP tissues (*n* = 3). Red arrow indicates change of *Pdgfra*. (**B** and **C**) Representative immunoblots (**B**) and immunofluorescence (**C**) of PDGFRα in E11.5 NP tissues (*n* = 4). (**D**) Relative mRNA expression of *Pdgfra* in NP tissues of embryos treated with or without LDN193189 (2 mg/kg/d, E8.5–E11.5, *n* = 4). (**E** and **F**) Cell proliferation assay (**E**) and wound-scratch assay (**F**) of NP cells treated with or without PDGFA (*n* ≥ 5). (**G** and **H**) Relative mRNA expression of *Pdgfra* in NP cells transfected with *Slc2a1* and *Hk2* overexpression plasmid (**G**, *n* = 3) or treated with sodium lactate (**H**, *n* = 3). (**I**–**K**) Results of Pan-Kla and H3K18la occupancy analysis by ChIP–qPCR at the promoter of *Pdgfra* in NP cells treated with or without 2-DG or sodium lactate (**I** and **J**, *n* ≥ 6). *Atcb* was used as a negative control to analyze Pan-Kla occupancy (**K**, *n* ≥ 7). (**L**) Results of pSmad1/5/9 occupancy analysis by ChIP–qPCR at the promoter of *Pdgfra* in control NP cells (*n* = 9). Antibodies against total H3 and preimmune rabbit IgG (Rb) were used as positive and negative controls, respectively (**L**, *n* = 9). For all panels, data are represented as mean ± SD. *P < 0.05; **P < 0.01; ***P < 0.001, unpaired 2-tailed Student’s *t* test (**B** and **K**) or 1-way ANOVA (**D**–**J** and **L**). Scale bars: 100 μm.

To identify downstream targets critical for midline facial development that might be regulated by histone lactylation, first, we revisited the bulk RNA-Seq data of E11.5 NP tissues to pick up genes known to associate with craniofacial midline abnormalities. Next, we isolated NP tissues from both control and ca-*Acvr1(A11)* mutants at E10.5 and quantified expression levels of genes identified through RNA-Seq. Among those, *Pdgfra* was the most significantly decreased transcript in ca-*Acvr1(A11)* NP tissues (Figure 4A). Western blot analyses and immunostaining confirmed reduced abundance of PDGFRα in the mesenchyme of ca-*Acvr1(A11)* NP tissues (Figure 4, B and C). In vivo administration of LDN193189 significantly rescued *Pdgfra* expression in ca-*Acvr1(A11)* NP tissues (Figure 4D). Notably, BMP-mediated regulation of *Pdgfra* histone lactylation in CNCCs was not restricted in the ca-*Acvr1(A11)* mutant line. Another ca-*Acvr1(L35)* mutant line (40), which displayed separation of nasal septum, also showed reduced histone lactylation and *Pdgfra* expression in NP tissues at E11.5 (Supplemental Figure 7, A–C). We further confirmed the effects of BMP signaling in regulating the lactylation/PDGFRA signaling cascade in vitro using the O9-1 cell line. Suppressing BMP signaling by LDN193189 reduced lactate levels and *Pdgfra* histone lactylation in O9-1 cells in vitro (Supplemental Figure 7, D–F).

Interestingly, the PDGFA/PDGFRα axis is essential for midfacial development, acting by regulating cell growth and migration. Furthermore, *Pdgfra* deletion causes a midline facial cleft in mice (33, 41). To directly assess whether ca-*Acvr1(A11)* mutant cells are functionally defective in transducing PDGFRα-mediated signaling, control and ca-*Acvr1(A11)* mutant NP cells were isolated and stimulated with PDGFA, a PDGFRα ligand (33). PDGFA significantly potentiated the growth and migration of control NP cells but not ca-*Acvr1(A11)* NP cells (Figure 4, E and F), suggesting that the levels of PDGFRα were too low for successful PDGFA signal transduction in mutant NP cells.

While the relationship between BMP signaling and PDGFRα remains unknown, histone lactylation has been reported to positively regulate gene expression (8). Indeed, either *Slc2a1* or *Hk2* overexpression or sodium lactate supplementation rescued *Pdgfra* expression in ca-*Acvr1(A11)* NP cells (Figure 4, G and H). To investigate whether *Pdgfra* is a target gene of histone lactylation in CNCCs, we next performed ChIP-qPCR of Pan-Kla and H3K18la at the promoter region of *Pdgfra* in control and ca-*Acvr1(A11)* NP cells. Importantly, the amounts of Pan-Kla and H3K18la at the promoter of *Pdgfra* were significantly lower in ca-*Acvr1(A11)* NP cells than in control cells (Figure 4, I and J). Additionally, exogenous 2-deoxy-d-glucose (2-DG) (to block glycolysis) significantly suppressed, whereas sodium lactate increased, the amount of histone lactylation at the promoter region of *Pdgfra* in control NP cells (Figure 4, I and J). In contrast, there was no alteration in histone lactylation at the promoter of the *Actb* gene (Figure 4K), which is not a target for lactylation (42). The ChIP-qPCR results showed that the amount of pSmad1/5/9 at the promoter region of *Pdgfra* was significantly lower than that of the total histone 3 (H3) (positive control) and comparable with a background level (rabbit IgG, Rb, negative control) in control NP cells (Figure 4L). Additionally, sodium lactate treatment restored *Pdgfra* expression without affecting pSmad1/5/9 levels in ca-*Acvr1(A11)* NP cells (Figure 4H and Supplemental Figure 4E). Together, these results indicate that *Pdgfra* is a target gene of lactylated histone, but not regulated by direct binding of pSmad1/5/9 to its promoter region, in CNCCs during midline facial development.

**Figure 5:**
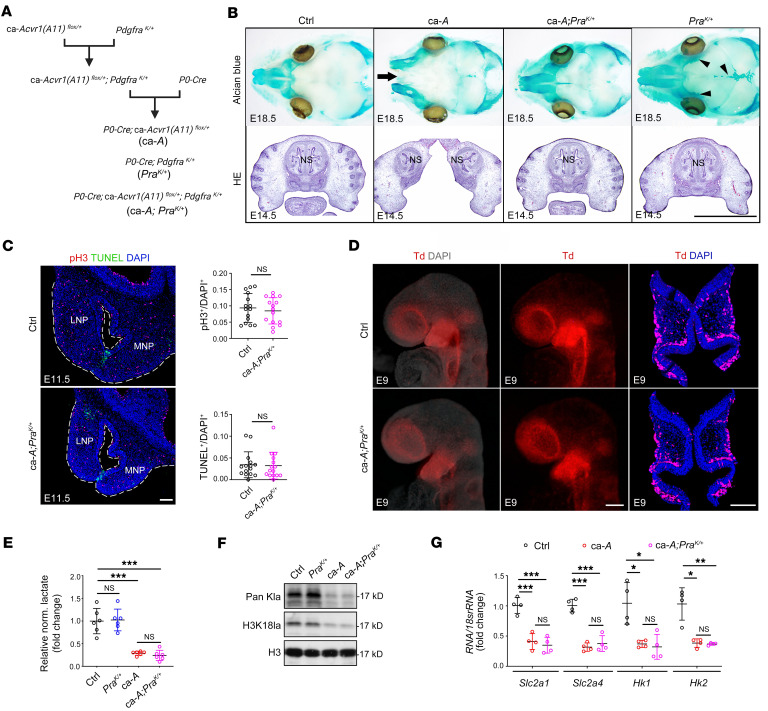
Reduced *Pdgfra* expression downstream of histone lactylation is critical for the midline facial defects of ca-*Acvr1(A11)* mutants. (**A**) Schematic showing the generation of transgenic mice with a constitutively activated *Pdgfra* allele *Pdgfra^K/+^*, *Pdgfra^K/+^* mutants (*Pdgfra^K/+^;P0-Cre*, abbreviated as *Pra^K/+^* in figures), and compound mutants (ca-*Acvr1(A11)^fl/+^*;*Pdgfra^K/+^;P0-Cre*, abbreviated as *ca-A;Pra^K/+^* in figures). (**B**) Whole-mount Alcian blue staining (upper) and H&E staining (lower) of embryo heads from control, ca-*Acvr1(A11)*, *Pdgfra^K/+^* mutants (*Pra^K/+^*), and compound mutants (*ca-A;Pra^K/+^*) at E18.5 and E14.5, respectively (*n* ≥ 6). Black arrow indicates midline facial cleft. Black arrowheads indicate increased cartilage formation in *Pdgfra^K/+^* mutants. (**C**) pH3 immunofluorescence (red) and TUNEL staining (green), and quantification results in E11.5 NP tissues (*n* = 6). (**D**) Lateral view of whole-mount embryo with DAPI staining (left) and without DAPI staining (middle) and coronal sections (right) of control and compound mutants labeled with *R26R^TdTomato^* (*n* = 5). (**E**) Relative normalized lactate levels in E11.5 NP tissues (*n* = 6). (**F**) Representative immunoblots of Pan-Kla and H3K18la in E11.5 NP tissues (*n* = 6). Results shown are from blots run contemporaneously. (**G**) Relative mRNA expression of *Slc2a1*, *Slc2a4*, *Hk1*, and *Hk2* in E11.5 NP tissues (*n* = 4). For all panels, data are represented as means ± SD. *P < 0.05; **P < 0.01; ***P < 0.001, unpaired 2-tailed Student’s *t* test (**C**) or 1-way ANOVA (**E** and **G**). Scale bars: 1 mm (**B**); 100 μm (**C** and **D**).

As the phenotype in ca-*Acvr1(A11)* mutants resembles that observed in mice with *Pdgfra* deletion (33, 41), we considered the possibility that PDGFRα may be a critical downstream effector of the BMP-signaling pathway that causes midline facial defects in ca-*Acvr1(A11)* mutants. As such, we superimposed a constitutively activated *Pdgfra* allele (*Pdgfra^K/+^*) (43) into ca-*Acvr1(A11)* mutants (*Pdgfra^K/+^;ca-Acvr1[A11]^fl/+^;P0-Cre*, abbreviated as compound mutants) (Figure 5A). Consistent with a previous report (44), constitutive activation of PDGFRα in NCCs increased cartilage formation in the skull (Figure 5B). Importantly, activation of PDGFRα signaling in ca-*Acvr1(A11)* mutants also resulted in significantly improved midline facial structures, including the appearance of a primary palate and a fused nasal septum (4 out of 7, Figure 5B), indicating that restoration of PDGFRα signaling ameliorated the midline facial defects of ca-*Acvr1(A11)* mutants. To determine whether restoring PDGFRα signaling likewise reversed the observed defects in cell migration and growth, compound-mutant embryos were superimposed with an *R26R^tdTomato^* reporter allele. Under these conditions, the compound mutants displayed a restoration of proliferative responses to control levels in tandem with a marked decrease in apoptosis at E11.5 (Figure 5C) along with rescued migratory activity at E9 (Figure 5D). However, superimposing a *Pdgfra^K/+^* allele failed to affect the levels of lactate and histone lactylation or the expression of glycolysis-related genes in the NP tissues of ca-*Acvr1(A11)* mutants (Figure 5, E–G). Collectively, these studies identify *Pdgfra* as a target of histone lactylation in CNCCs and indicate that BMP signaling–dependent regulation of histone lactylation directs CNCC development.

In addition to craniofacial morphogenesis, defects were identified in other NCC-derived tissues in ca-*Acvr1(A11)* mutants, such as in cardiac neural crest–derived tissues of the heart (27) and trunk neural crest–derived dorsal root ganglion (DRG) of the peripheral nervous system in association with elevated pSmad1/5/9 and cell death (Supplemental Figure 8, A–D). Similarly, elevated BMP signaling downregulated the histone lactylation and PDGFRA expression in cardiac and trunk neural crest derivatives in vivo (Supplemental Figure 8, E and F). We also confirmed these facts in vitro using trunk NCCs (Supplemental Figure 8, G and H). These facts suggest a broader role for the BMP-histone lactylation cascade in various NCC-derived tissues.

### BMP signaling suppresses histone lactylation by potentiating p53 activity.

**Figure 6:**
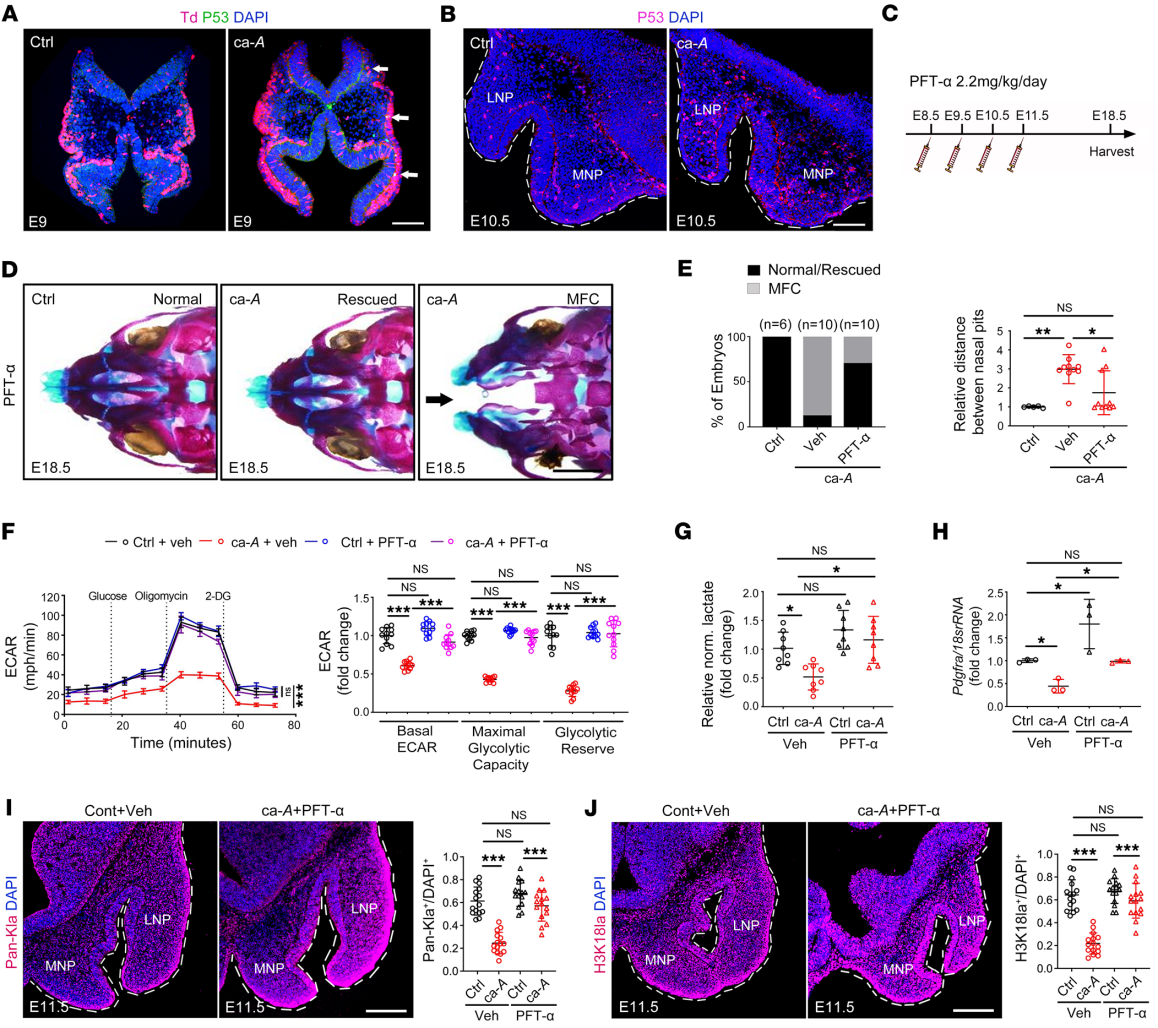
BMP signaling suppresses lactate-derived histone lactylation via potentiating p53 activity. (**A** and **B**) p53 immunofluorescence in E9 migrating CNCCs (**A**, *n* = 6) and E10.5 mesenchymal cells of NP tissues (**B**, *n* = 6). White arrows indicate p53-positive cells in CNCCs. (**C**–**E**) Inhibitor injection schedule (**C**), whole-mount skeletal staining (**D**), quantification of the embryo number (**E**, left), and distance between nasal pits (**E**, right) of embryos treated with or without PFT-α (2.2 mg/kg/d, E8.5–E11.5, *n* ≥ 6). Black arrow indicates midline facial cleft. (**F**) ECAR levels, calculated glycolytic flux, and glycolytic capacity in control and ca-*Acvr1(A11)* NP cells treated with or without PFT-α (*n* = 11). (**G**) Relative normalized lactate levels in control and ca-*Acvr1(A11)* NP cells treated with or without PFT-α (*n* = 8). (**H**) Relative mRNA expression of *Pdgfra* in control and ca-*Acvr1(A11)* NP cells treated with or without PFT-α (*n* = 3). (**I** and **J**) Pan-Kla (red, **I**) and H3K18la (red, **J**) immunofluorescence and quantification results in NP tissues of embryos treated with or without PFT-α administration (2.2 mg/kg/d, E8.5–E11.5, *n* = 6). For all panels, data are represented as means ± SD. *P < 0.05; **P < 0.01; ***P < 0.001, 1-way ANOVA (**E**–**J**). Scale bars: 100 μm (**A**, **B**, **I**, and **J**); 1 mm (**D**).

We next investigated how BMP/Smad signaling suppresses glycolytic activity and histone lactylation in CNCCs during midfacial development. While BMP signaling regulates Glut1 expression via mTORC1/HIF1α signaling in skeletal development (36), we did not detect significant differences in the expression of HIF-1α and the mTORC1 downstream effector pS6 between control and mutant NP tissues (Supplemental Figure 9, A and B). Autophagy, another important regulator of glucose metabolism (45), was not altered in the NP tissues of ca-*Acvr1(A11)* mutants, as the protein abundance of LC3-II and P62 was comparable between the controls and mutants (Supplemental Figure 9C). The difference between our study and previous reports may be a cell-type–difference outcome. We have previously demonstrated that BMP/Smad signaling increases p53 activity by suppressing its degradation (26), and of note, p53 can affect glycolytic activity (46). Interestingly, inappropriate p53 activation in the neural crest was recently reported to cause midline facial cleft (47). Although there was no obvious change of *Trp53* at the gene expression level in ca-*Acvr1(A11)* NP tissues (Supplemental Figure 9A), p53 protein levels were increased in migrating CNCCs at E9 and in the mesenchyme of NP tissues at E10.5 (Figure 6, A and B, and Supplemental Figure 9D). In vivo administration of LDN193189 reduced p53 protein levels in ca-*Acvr1(A11)* NP tissues (Supplemental Figure 9E). Importantly, suppressing p53 activity using the specific p53 inhibitor pifithrin-α (PFT-α) significantly rescued midline facial structures of ca-*Acvr1(A11)* mutants with a fused nasal septum and reduced distance between nasal pits (Figure 6, C–E). Using primary NP cells, we also found that PFT-α successfully restored the expression of glycolysis-associated genes, glycolytic capacity, lactate levels, and *Pdgfr*α expression in ca-*Acvr1(A11)* NP cells (Supplemental Figure 9F and Figure 6, F–H). Coincident with these above changes, PFT-α–treated ca-*Acvr1(A11)* embryos display comparable protein levels of Pan-Kla and H3K18la in the NP mesenchymal tissues relative to controls (Figure 6, I and J). We further determined whether p53 activation functions upstream of glycolysis and histone lactylation to regulate midline facial development of ca-*Acvr1(A11)* mutants by treating pregnant females with PFT-α and 2-DG. Skeletal staining showed that 2-DG significantly abrogated the restorative effects of PFT-α on midline facial development of ca-*Acvr1(A11)* mutants (Supplemental Figure 9, G and H, and Figure 6, C–E). Notably, although 2-DG alone only caused minor defects with a bifid nasal tip in a subset of control embryos, the same dose of 2-DG caused a more severe phenotype in ca-*Acvr1(A11)* mutants (Supplemental Figure 9H). Together, these data support a model wherein excessive BMP signaling suppresses histone lactylation by potentiating p53 activity, thereby inducing midline facial defects.

## Discussion

Neural crest cells display the metabolic adaptation known as the Warburg effect where enhanced glycolytic activity is required for migratory responses in vertebrate embryos (35). Nevertheless, the complex mechanisms underlying coordinated regulation of neural crest cells at the levels of gene expression, signaling, and metabolism during embryonic development remain poorly characterized. Our studies uncover a signaling metabolic/epigenetic transcascade that operates during craniofacial morphogenesis wherein the finely tuned control of BMP signaling is a required determinant of glycolytic lactate production and the subsequent epigenetic control of *Pdgfra* histone lactylation, thereby regulating CNCC development in vitro and in vivo.

In response to the binding of BMP ligands, type I receptors of BMPs are transphosphorylated by type II BMP receptors, which consequently phosphorylate Smad signal transducers to propagate downstream signaling (14). We and others have reported that an appropriate amount of BMP signaling is required for craniofacial and skeletal morphogenesis by controlling the osteogenic, chondrogenic, and neurogenic differentiation of stem cells (15, 24, 25, 48, 49). We recently reported that augmenting BMP signaling using the ca-*Acvr1(L35)* line (40) directs CNCCs to a chondrogenic fate (50). Contrasting with these findings, our present studies using ca-*Acvr1(A11)* mice reveal a function of neural crest cell–specific activation of BMP/Smad signaling in midline facial development, which acts by regulating CNCC migration and growth while leaving differentiation potential intact. The relevance of enhanced BMP signaling for midline facial development is underscored by the fact that *Noggin* loss-of-function mutations lead to bifid nasal pits in humans (22). We have reported that ca-*Acvr1(A11)* mutants investigated in the present study show phenotypes distinct from another ca-*Acvr1(L35)* line, which we determined by crossing with 3 different Cre lines (*P0-Cre*, *Prrx1-Cre*, and *Nfatc1-Cre*) (27). The explanations for the phenotypic differences between ca-*Acvr1(A11)* and ca-*Acvr1(L35)* are found in the levels of BMP/Smad signaling and/or the differences in cell types that express the transgene (27). We noticed that neural crest–specific ca-*Acvr1(L35)* mutants (ca-*Acvr1(L35)^fl/+^;P0-Cre*) showed a separation of nasal septum, while the structure of nasal cavity was disorganized, suggesting ca-*Acvr1(A11)* is more advantageous than ca-*Acvr1(L35)* in reflecting the developmental nature of midline facial cleft. Notably, we also confirmed the suppression effects of BMP signaling on the histone lactylation/PDGFRA signaling cascade in ca-*Acvr1(L35)* mutants in vivo. Together with the in vitro study using O9-1 cells treated with BMP ligands, these facts demonstrate that suppression of the lactylation/PDGFRA cascade is a common nature of enhanced BMP signaling in CNCCs.

Each BMP type I receptor has unique functions in CNCCs and craniofacial development. Mice lacking *Acvr1* in NCCs (*Acvr1^fl/fl^;Wnt1-Cre*) display craniofacial defects that are quite different from those of mice lacking *Bmpr1a* in NCCs (*Bmpr1a^fl/fl^;Wnt1-Cre*) (25, 48, 49). Transgenic mouse models with constitutive activation of *Bmpr1a* or *Acvr1* in NCCs also confirm the receptor specificity of the phenotype (24, 26, 50). This notion is also supported by the fact that superimposing of heterozygous null mutation of *Bmpr1a*, but not *Bmpr1b* or *Acvr1*, reduces BMP/Smad signaling and rescues the premature suture fusion of ca-*Bmpr1a;P0-Cre* mutants (51). The receptor-specific phenotypes may be explained by (a) each BMP type I receptor having its unique expression pattern in different subpopulations of CNCCs during development, and/or (b) each BMP type I receptor mediating different levels of BMP signaling in CNCCs (51, 52). Notably, BMP ligands, including *Bmp2*, *Bmp4*, *Bmp5*, and *Bmp7*, were observed in the epithelia of facial processes at E9.5, while only *Bmp5* and *Bmp7* were expressed in the epithelia and mesenchyme of NPs at E10.5 (53, 54), suggesting BMP5 and BMP7 can serve as the potential source of BMP signal during midfacial morphogenesis. *Bmp5* and *Bmp7* double-knockout mice have a reduced size of their branchial arches, including facial processes, at E10.5, while neural crest–specific deletion of *Bmp7* presents with midfacial hypoplasia and nasal septum deviation (54–56). The specific function of BMP5 and BMP7 in epithelia or mesenchyme during midfacial morphogenesis remains to be clarified.

BMP signaling regulates multiple steps of NCC biology, including NCC delamination and migration, in a highly context-dependent manner. NCC delamination/initial induction is enhanced in embryos lacking BMP antagonists (*Noggin* and *Chordin* double-null mutants), but normal in embryos with mutations in *Bmp2*, *Bmpr1a*, or *Acvr1* (49, 57–60). NCC migration is affected in embryos with *Bmp2*-null mutation or *Noggin* and *Chordin* double-null mutants, but normal in embryos with ablation of *Bmpr1a* or *Acvr1* in neural crest cells (49, 57–60). In the present study, we showed that enhanced BMP signaling via constitutively active *Acvr1* caused a retardation of CNCC migration without affecting CNCC delamination. The potential explanation for the context-dependent function of BMP signaling in NCC delamination and migration included that (a) each BMP ligand or receptor has its unique expression pattern in NCCs and (b) each BMP ligand or receptor mediates different levels of downstream events of BMP signaling.

Glycolysis has been proposed as playing a necessary role in supporting the delamination and migration of chicken neural crest cells (35). Recently, BMP signaling has been shown to activate glycolysis via mTORC1 and HIF-1α in chondrocytes (36). In our study, we found that augmented BMP signaling blocks glycolytic activity and lactate production in CNCCs by potentiating p53 activity during midline facial morphogenesis. The different effect of BMP signaling on glycolysis activity could be due to different ways of signal activation, i.e., sustained (our case) versus ligand-dependent activation. An alternative explanation is differences in cell types and/or biological context, i.e., immature cells (our case) versus terminally differentiated cells. Each BMP ligand is known to have its unique yet pleiotropic functions in development and human disease (13). As an example of the difference in cell context, it has been reported that BMP4 can induce commitment of mesenchymal stem cells to preadipocytes of both white and brown lineage, whereas BMP4 signaling during the terminal differentiation phase impairs the acquisition of a mature brown adipocyte phenotype in favor of a more white-like phenotype (61).

Metabolite intermediates can directly modulate cellular function at diverse levels, especially via epigenetic modifications (8, 17). Histone lactylation is a recently identified posttranslational modification that regulates gene expression during embryonic stem cell pluripotency and induction as well as macrophage immune responses (10). Indeed, we found that elevated BMP/Smad signaling disrupts histone lactylation, rather than histone acetylation or methylation, thus causing a reduction in CNCC *Pdgfra* expression during midline facial development. Of note, supplementation of exogenous lactate enhanced histone lactylation levels and *Pdgfra* expression, resulting in the rescue of both CNCC function and midline facial defects in ca-*Acvr1(A11)* mutants. Therefore, our study identifies the functional relevance of histone lactylation during embryonic development in vitro and in vivo.

PDGFRα signaling, together with signaling of its ligand, PDGFA, has been suggested as functioning as a chemotactic cue for NCCs (33). Perturbations of PDGFRα signaling in mouse and zebrafish lead to severe defects in CNCC-derived tissues, including midline facial structures (33, 62). Our results show a reduction of *Pdgfra* in ca-*Acvr1(A11)* mutants. Using a constitutively active form of knockin allele for *Pdgfra*, our results further show that activating *Pdgfra* in ca-*Acvr1(A11)* mutants rescues the defects in the migration and growth of CNCCs as well as the midline facial-defects phenotype. These results demonstrate that PDGFRα-mediated signaling is essential for midline facial morphogenesis downstream of BMP signaling. We further identified the expression of *Pdgfra* as being regulated by glycolysis-mediated histone lactylation. There is a possibility that other mechanisms regulate *Pdgfra* expression; however, our data showing that (a) lactate supplementation or overexpression of glycolysis mediators increases *Pdgfra* expression in ca-*Acvr1(A11)* mutants and that (b) *Pdgfra* is a direct target gene of histone lactylation strongly suggest that BMP signaling functions via histone lactylation to regulate *Pdgfra* expression during midfacial development. On the other hand, *Pdgfra* activation does not affect lactate production or glycolysis mediator expression, suggesting that *Pdgfra* functions downstream of glycolytic lactate production, but not upstream. Our findings therefore identify crosstalk between BMP and PDGFRα signaling that oversees the fine-tuned regulation of CNCC behavior and craniofacial morphogenesis.

Nevertheless, BMP-regulated histone lactylation may well extend its control beyond *Pdgfra* expression. Previous investigation in other cell types reveals that histone lactylation regulates expression of several genes, such as *Pdgfrb*, *Thbs1*, *Mycn*, *Arg1*, and *Vegfa*, in other cell types (8, 44, 63–68). Our RNA-Seq of ca-*Acvr1(A11)* mutants showed reduced levels of *Pdgfrb* and *Thbs1* expression. PDGFRβ and PDGFRα are codistributed in the craniofacial mesenchyme of midgestation mouse embryos, and PDGFRα and PDGFRβ double-homozygous mutant embryos exhibit more severe facial-clefting phenotypes than those observed in single-mutant embryos. However, *Pdgfrb* may not be a determinant for the phenotype in ca-*Acvr1(A11)* mutants because (a) ablation of *Pdgfrb* alone in the neural crest lineage did not result in midline facial defects (69) and (b) our results clearly showed that *Pdgfra* activation rescued the midfacial phenotype of ca-*Acvr1(A11)* mutants. Thrombospondin 1 (THSB-1) encoded by *Thbs1* has concentration-dependent effects on migration, adhesion, and proliferation of chicken neural crest cells in vitro (70). The potential function of THSB-1 in midline facial development remains to be clarified. Despite a possibility of other targets of lactylated histones as downstream of BMP signaling in CNCCs, our data clearly demonstrate that *Pdgfra* is one of the major downstream target genes of the BMP-lactylation cascade that results in midline facial clefts of ca-*Acvr1(A11)* mutants.

In the present study, we demonstrate a mechanism by which histone lactylation critically regulates *Pdgfra* expression as a downstream target of BMP signaling in CNCCs. We propose the signaling cascade BMP/p53/histone lactylation/PDGFRA in regulating CNCC behaviors and midline facial development. The identified BMP signaling/metabolic/epigenetic transcascade can serve as a conceptual framework for understanding the complex interactions between genetic and metabolic cues during embryonic development. Extending these observations, these findings suggest that elevated BMP signaling and reduced lactate levels could serve as biomarkers for identifying individuals with an increased risk of having a child with congenital craniofacial malformations. In addition, our molecular mechanisms will be useful for interpreting the results of large-scale population-based genetic studies aimed at identifying inherited risk factors for craniofacial malformations. Finally, in the longer term, our results may identify potential safe and effective maternal nutritional and therapeutic interventions that reduce the risk of offspring developing these congenital malformations.

## Methods

### Sex as a biological variable.

Our study examined male and female mice; no sex-biased outcomes were observed. Thus, we combined data from both sexes for all experiments.

### Animals.

ca-*Acvr1^fl/+^* line A11, B6;129S7-Tg (CAG-lacZ,-ACVR1*,-EGFP)2Mis (27), and ca-*Acvr1^fl/+^* line L35 mice (40) were generated in our laboratory. *P0-Cre* mice, C57BL/6J-Tg(P0-Cre)94Imeg (ID 148), were provided by the Center for Animal Resources and Development, Kumamoto University, Kumamoto City, Japan. *Pdgfra^K/+^* mice were provided by Lorin E. Olson at the Oklahoma Medical Research Foundation. The PDGFRα^K^ protein has a point mutation, D842V, which renders it constitutively active (43). *Wnt1-Cre*, *Rosa26-loxP-stop-loxP-tdTomato* (*R26R^tdTomato^*, JAX007914) mice were obtained from the Jackson Laboratory. ca-*Acvr1(A11)^fl/+^* mice were crossed with *P0-Cre* transgenic mice to obtain control (ca-*Acvr1[A11]*^+/+^;*P0-Cre*) and ca-*Acvr1(A11)* mutant (ca-*Acvr1[A11]^fl/+^*;*P0-Cre*) mice. ca-*Acvr1(A11)^fl/+^* mice were crossed with *Wnt1-Cre* transgenic mice to obtain control (ca-*Acvr1[A11]*^+/+^;*Wnt1-Cre*) and ca-*Acvr1(A11)^Wnt1-Cre^* mutant (ca-*Acvr1[A11]^fl/+^*;*Wnt1-Cre*) mice. ca-*Acvr1(L35)^fl/+^* mice were crossed with *P0-Cre* transgenic mice to obtain control (ca-*Acvr1[L35]*^+/+^;*P0-Cre*) and ca-*Acvr1(L35)* mutant (ca-*Acvr1[L35]^fl/+^*;*P0-Cre*) mice. In vivo fate mapping of neural crest–derived cells was performed on mice additionally carrying the *R26R^tdTomato^* allele. All mice were maintained in a mixed background and were used and genotyped as previously described (50). Embryos were collected from pregnant mice. Embryonic ages were determined by the day when the vaginal plug was discovered, which was designated E0.5. Mutant and control embryos from E8.5–E11.5 were age matched by counting the number of somites. Embryonic tails were subjected to DNA extraction for genotyping as previously described (50). For genotyping primers, see Supplemental Table 1.

### Statistics.

Graphs were generated and statistical analyses were performed using Prism. All values are expressed as means ± SD. Unpaired, 2-sided Student’s *t* test was used to analyze the differences between 2 groups, while 1-way ANOVA with Bonferroni’s correction was used to evaluate differences among multiple comparisons. All experiments were repeated at least 3 times, and representative experiments are shown. Differences were considered significant at *P* < 0.05. In each experiment, the sample size was determined on the basis of our prior knowledge of the variability of the experimental output. No randomization was performed in any of the experiments. All inclusion/exclusion criteria were preestablished, and no samples or animals were excluded from the analysis. The investigators were blinded to allocation during the experiments and outcome assessments.

### Study approval.

All animal handling and procedures were carried out in compliance with University of Michigan and Wuhan University guidelines and approved by the IACUC at the University of Michigan (#PRO00007715 and 00009613) and the IACUC of the Wuhan University Center for Animal Experiments (#WP20210535).

### Data and materials availability.

The raw data, analytic methods, and study materials are described in full in Supplemental Methods. Values for all data points in graphs are reported in the Supporting Data Values file. Information for qRT-PCR primers, ChIP-qPCR primers, and antibodies used in this report appears in Supplemental Tables 2–4, respectively. Metabolomics data have been deposited to Mendeley (https://data.mendeley.com/datasets/cr5dfc4mnk/1). RNA-Seq data have been deposited to the NCBI’s Gene Expression Omnibus database (GEO GSE247729).

## Author contributions

JY and YM designed and supervised the project, analyzed data, wrote the manuscript, and finalized the manuscript. LZ designed the project, analyzed data, wrote the manuscript, and finalized the manuscript. LEO provided key materials and relevant advice. JY, LZ, HU, HP, MT, QY, SAH, and YM performed experiments, analyzed data, and provided relevant advice. The order of the co–first authors’ names reflects person-hours and contributions to the writing of the manuscript, preparation of figures, and study design. All authors approved the final version.

## Supplementary Material

Supplemental data

Unedited blot and gel images

Supplemental table 1

Supplemental table 2

Supplemental table 3

Supplemental table 4

Supporting data values

## Version 1 03/11/2024

In-Press Preview

## Version 2 04/15/2024

Electronic publication
