# Supplementary material for: A BMP-controlled metabolic/epigenetic signaling cascade directs midfacial morphogenesis
Source: J Clin Invest. 2024 Mar 11;134(8):e165787. doi: 10.1172/JCI165787 (PMC11014657; doi:10.1172/JCI165787)
Supplement: Supplemental data [file jci-134-165787-s168.pdf]

TITLE: A BMP-controlled metabolic-epigenetic signaling cascade directs midfacial morphogenesis

AUTHORS: Jingwen Yang[1,2#*], Lingxin Zhu[1,3#], Haichun Pan[2], Hiroki Ueharu[2], Masako Toda[2], Qian Yang[1], Shawn A. Hallett[2], Lorin E. Olson[4], Yuji Mishina[2*]

AFFILIATIONS:

[1] State Key Laboratory of Oral & Maxillofacial Reconstruction and Regeneration, Key Laboratory of Oral Biomedicine Ministry of Education, Hubei Key Laboratory of Stomatology, School & Hospital of Stomatology, Wuhan University, Wuhan, Hubei, China.

[2] Department of Biologic and Materials Sciences, School of Dentistry, University of Michigan, Ann Arbor, Michigan, USA.

[3] Life Sciences Institute, University of Michigan, Ann Arbor, Michigan, USA.

[4] Cardiovascular Biology Research Program, Oklahoma Medical Research Foundation, Oklahoma City, Oklahoma, USA.

# Jingwen Yang and Lingxin Zhu contribute equally.

*To whom correspondence should be addressed:

Jingwen Yang, email address: jingwen.yang@whu.edu.cn, telephone: (+86)027-87686211, address: 237 Luoyu Road, Wuhan 430079, Hubei, China

Yuji Mishina, email address: mishina@umich.edu (Y.M.), telephone: (+1)734-763-5579, address: 4222A Dental, 1011 N. University Ave, Ann Arbor, Michigan, 48109-1078, USA.

ONLINE SUPPLEMENTAL FIGURES

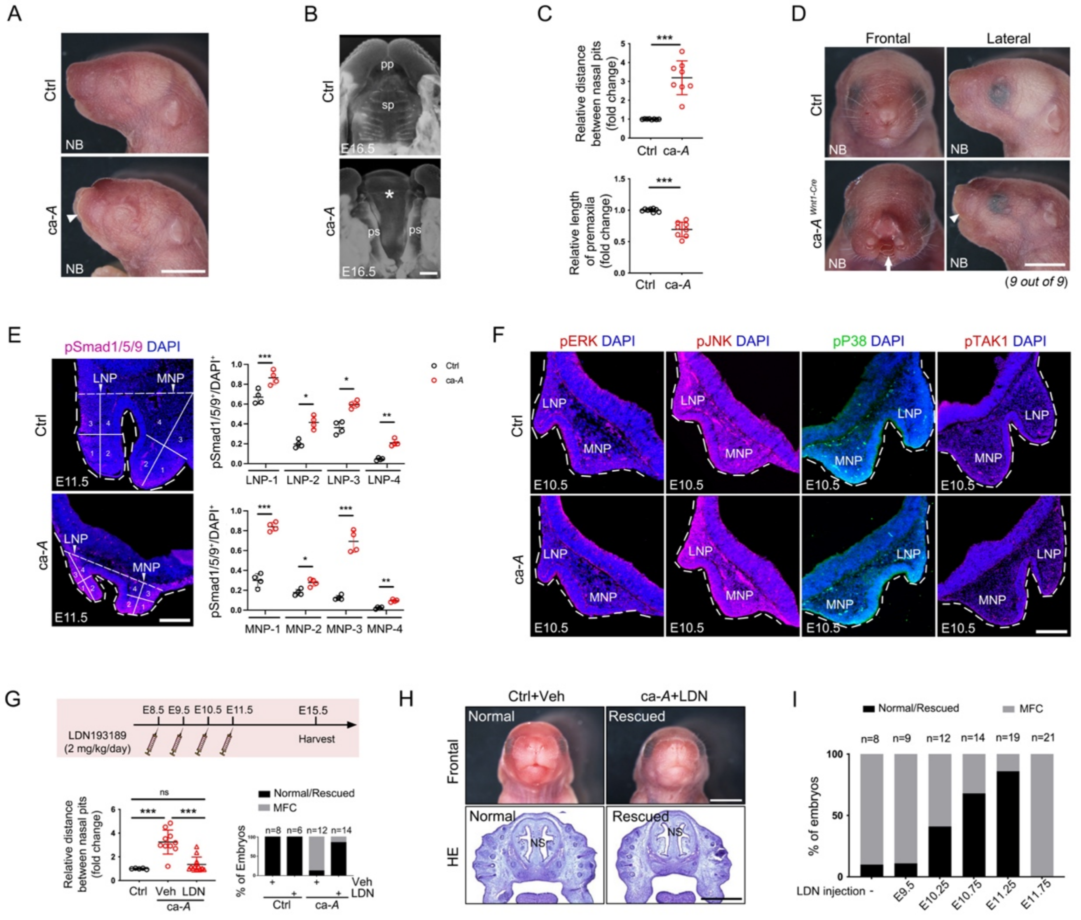

Figure S1 ca-ACVR1 in NCCs increases of BMP-Smad signaling causing midline facial defects.

(**A**) Lateral view of control and *ca-Acvr1(A11)* embryos (*P0-Cre;*ca-*Acvr1(A11)*, abbreviated as ca-*A* in figures) at newborn (NB) stage (*n* = 11). White arrowhead indicates short snouts (100%). (**B**) Ventral view of DAPI-stained E16.5 embryos from which the lower jaw was removed (*n* = 6). The white asterisk indicates the absence of the primary palate. (**C**) Quantification results of the distance between nasal pits and the length of the premaxilla (*n* = 8). (**D**) Gross morphology of control and *Wnt-Cre;ca-Acvr1(A11)* (abbreviated ca-$A^{Wnt1\text{-}Cre}$) embryos at NB stage (*n* = 9). The white arrow indicates midline facial clefting. The white arrowhead indicates a short snout.

(**E**) Quantification and statistical analysis of pSmad1/5/9-positive cells in eight subregions of NPs (*n* = 4). (**F**) pERK (red), pJNK (red), pP38 (green) and pTAK1 (red) immunofluorescence of E10.5 NP tissues (*n* = 3). (**G**) LDN193189 injection schedule (upper), quantification of the relative distance between nasal pits and embryo number (lower) of control and *ca-Acvr1(A11)* mutants treated with or without LDN193189 (2 mg/kg/day, E8.5-E11.5, $n \geq 6$). (**H**) Representative frontal view and hematoxylin and eosin (HE) staining of frontal sections of control and *ca-Acvr1(A11)* mutants treated with or without LDN193189 (2 mg/kg/day, E8.5-E11.5, $n \geq 6$). (**I**) Quantification of *ca-Acvr1(A11)* embryo number treated with or without single injection of LDN193189 at indicated stage (2 mg/kg, one injection, $n \geq 8$). For all panels, data are presented as mean ± SD. ***$P < 0.001$, **$P < 0.01$, *$P < 0.05$, ns no significant difference, determined via unpaired 2-tailed Student's *t* test (C, E) or one-way ANOVA (G). Scale bars: 2 mm (A, B, D), 100 µm (E, F), 500 µm (G). MNP, medial nasal processes; LNP, lateral nasal processes; NS, nasal septum, pp, primary palate; sp, secondary palate; ps, palate shelf.

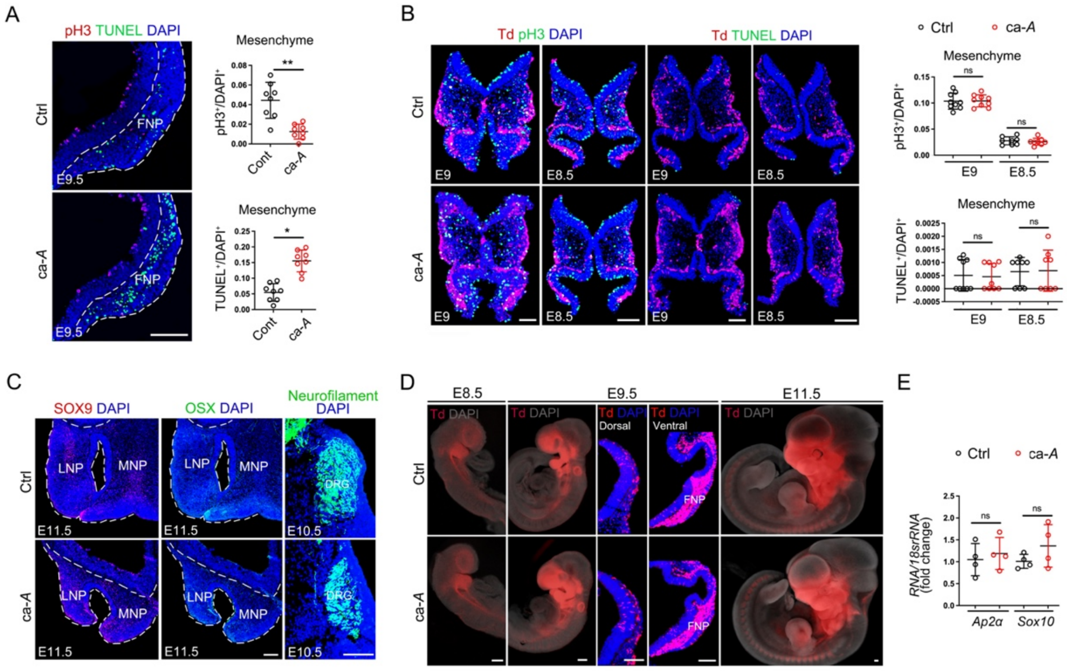

Figure S2 Enhanced BMP-Smad signaling in CNCCs regulates cell growth, differentiation, and migration in a stage-dependent manner.

(**A**) pH3 immunostaining (red), TUNEL staining (green) in E9.5 FNP (*n* = 8). (**B**) pH3 immunostaining (green, left), TUNEL staining (green, right) in CNCCs (red, *R26R*^{TdTomato}-positive) at E9 and E8.5 ($n \geq 8$). (**C**) SOX9 (red, left), OSX (green, middle) immunofluorescence of NP tissues and Neurofilament (green, right) immunofluorescence of cranial DRG at E11.5 (*n* = 3). (**D**) CNCCs (red, *R26R*^{TdTomato}-positive) distribution in control and *ca-Acvr1(A11)* embryos at indicated stages ($n \geq 4$). (**E**) Relative mRNA expression of *Ap2α* and *Sox10* in control and *ca-Acvr1(A11)* embryos at E8.5 (*n* = 4). For all panels, data are presented as mean ± SD. **$P < 0.01$, *$P < 0.05$, ns no significant difference, determined via unpaired 2-tailed Student's *t* test (A, B, E). Scale bars: 100 μm. FNP, frontonasal prominence; DRG, dorsal root ganglion; MNP, medial nasal processes; LNP, lateral nasal processes.

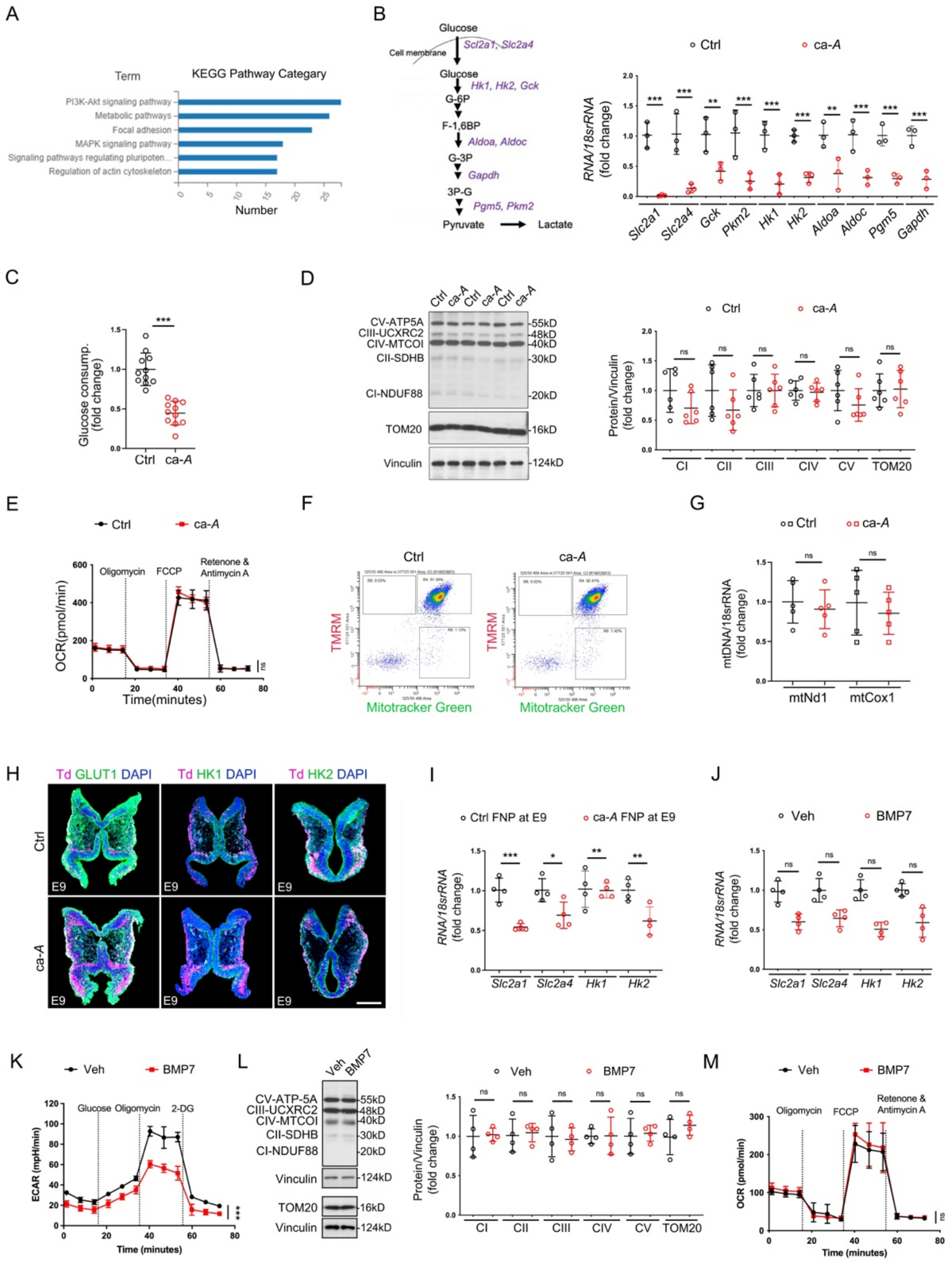

Figure S3 Enhanced BMP signaling suppresses glycolysis but not mitochondrial respiration in nasal processes.

(**A**) The number of differentially expressed genes and names of the most top signaling pathways affected in E11.5 ca-*Acvr1(A11)* NP tissues compared with controls by KEGG analysis (n = 3). (**B**) Relative mRNA expression of key mediators of

glycolysis, such as *Slc2a1*, *Slc2a4*, *Gck*, *Pkm2*, *Hk1*, *Hk2*, *Aldoa*, *Aldoc*, *Pgm5* and *Gapdh* in E10.5 NP tissues (n = 3). (**C**) Glucose uptake of NP cells (*n* = 11). (**D**) Representative immunoblots and quantification results of OXPHOS complexes and TOM20 in E10.5 NP tissues (*n* = 6). (**E**) Measurement of oxygen consumption rate (OCR) in NP cells (*n* = 5). (**F**) Flow cytometry results of MitoTracker Green (green) and TMRM (red) in NP cells (*n* = 3). (**G**) Relative DNA levels of mtNd1 and mtCox1 in E10.5 NP tissues (*n* = 5). (**H**) GLUT1 (green, left), HK1 (green, middle), and HK2 (green, right) immunofluorescence in CNCCs (red, $R26R^{TdTomato}$-positive) at E9 (*n* = 6). (**I**) Relative mRNA expression of *Slc2a1*, *Slc2a4*, *Hk1*, and *Hk2* in embryo heads at E9 (*n* = 4). (**J**) Relative mRNA expression of *Slc2a1, Slc2a4, Hk1, and Hk2* in O9-1 cells treated with or without BMP7 (*n* = 4). (**K**) Measurement of ECAR in O9-1 cells treated with or without BMP7 (*n* = 6). (**L**) Representative immunoblots and quantification of OXPHOS complexes and TOM20 in O9-1 cells treated with or without BMP7 (*n* = 4). Results shown are from blots run contemporaneously. (**M**) Measurement of OCR in O9-1 cells treated with or without BMP7 (*n* = 6). For all panels, data are presented as mean ± SD. ***$P < 0.001$, **$P < 0.01$, *$P < 0.05$, ns no significant difference, determined via unpaired 2-tailed Student's *t* test (B-E, G, I-M). Scale bars: 100 μm (H). See complete unedited blots in the supplemental material.

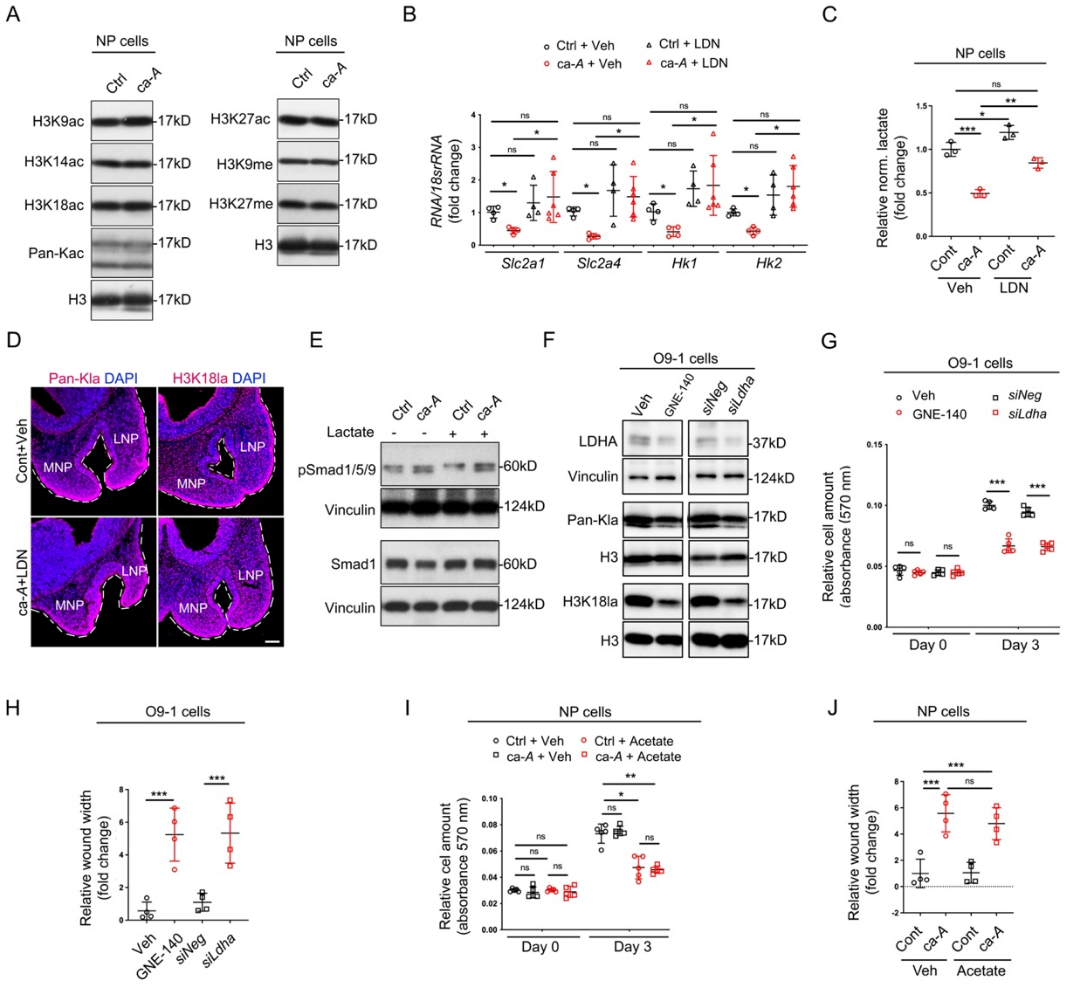

Figure S4 Histone lactylation, but not acetylation or methylation, regulates the growth and migration of CNCCs.

(**A**) Representative immunoblots of acetylation markers (H3K9ac, H3K14ac, H3K27ac, Pan-Kac) and methylation markers (H3K9me, H3K27me) in E10.5 NP tissues (n = 6). Results shown are from blots run contemporaneously. (**B, C**) Relative mRNA expression of *Slc2a1*, *Slc2a4*, *Hk1*, and *Hk2* (B) and relative normalized lactate levels (C) in NP tissues of embryos treated with or without LDN193189 (2 mg/kg/day, E8.5-E11.5, $n \geq 3$). (**D**) Representative immunostaining of Pan-Kla (red, left) and H3K18la (red, right) in NP tissues of embryos treated with or without LDN193189 (2 mg/kg/day, E8.5-E11.5, $n \geq 3$). (**E**) Representative immunoblots of pSmad1/5/9, Smad1 in NP cells treated with or without sodium lactate (n = 3). Results

shown are from blots run contemporaneously. (**F**) Representative immunoblots of LDHA, Pan-Kla and H3K18la in O9-1 cells treated with GNE-140 for 24 hr or transfected with *Ldha siRNA* (n = 6). Results shown are from blots run contemporaneously. (**G, H**) Quantification of cell proliferation assay (G, n = 5) and wound scratch assay (H, n = 4) results of O9-1 cells treated with GNE-140 or knockdown of *Ldha*. (**I, J**) Quantification of cell proliferation assay (I, n = 5) and wound scratch assay (J, n = 4) results of NP cells treated with sodium acetate. For all panels, data are presented as mean ± SD. ***P < 0.001, **P < 0.01, *P < 0.05, ns no significant difference, determined via unpaired 2-tailed Student's t test (G, H) or one-way ANOVA (B, C, I, J). Scale bars: 100 μm (D). See complete unedited blots in the supplemental material.

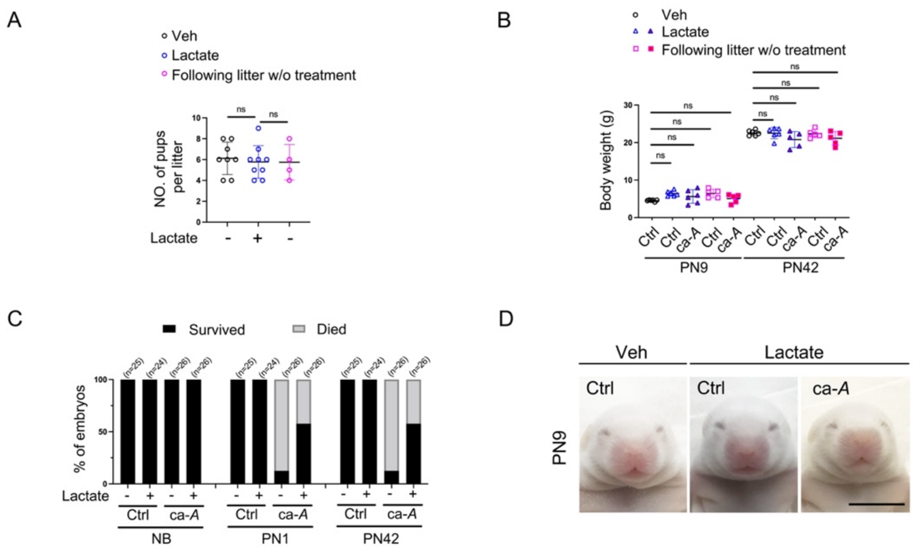

Figure S5 The rescued ca-*Acvr1(A11)* mutants by exogenous sodium lactate can survive postnatally.

(**A**) Litter size (number of pups at NB) of females treated with vehicle (Veh, n=8) or sodium lactate (lactate, n=9). The females in the lactate-treated group were bred again and their litter size was measured (following litter w/o treatment, n=4). (**B**) Body weight of control and ca-*Acvr1(A11)* mutant pups treated with veh or sodium lactate ($n \geq 6$). The females in the lactate-treated group were bred again without treatment and their pups' body weights were measured (following litter w/o treatment, $n \geq 24$). (**C**) The ratio of survived pups in control or mutants treated with or without sodium lactate at NB, PN1, and PN42 ($n \geq 24$). (**D**) Frontal view of PN9 pups treated with or without sodium lactate ($n \geq 5$). ns no significant difference, determined via one-way ANOVA (A, B). Scale bar: 1 mm (D).

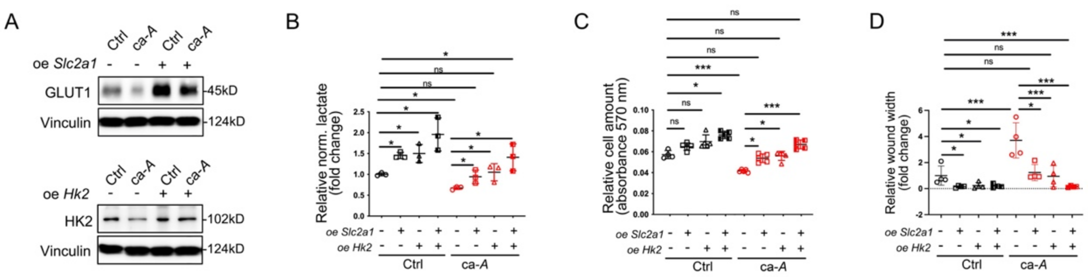

Figure S6 Restoring expression of glycolysis mediators ameliorates the growth and migration defects of ca-*Acvr1(A11)* NP cells.

(**A**) Representative immunoblots of GLUT1 and HK2 in NP cells transfected with *Slc2a1* or *Hk2* plasmid for 24 hr (n = 3). Results shown are from blots run contemporaneously. (**B**) Relative normalized lactate levels in NP cells transfected with *Slc2a1* or *Hk2* plasmid for 24 hr (n = 3). (**C, D**) Quantification of cell proliferation assay (C) and wound scratch assay (D) of NP cells transfected with *Slc2a1* or *Hk2* plasmid for 24 hr ($n \geq 4$). For all panels, data are presented as mean ± SD. ***$P < 0.001$, *$P < 0.05$, ns no significant difference, determined via one-way ANOVA (B-D). See complete unedited blots in the supplemental material.

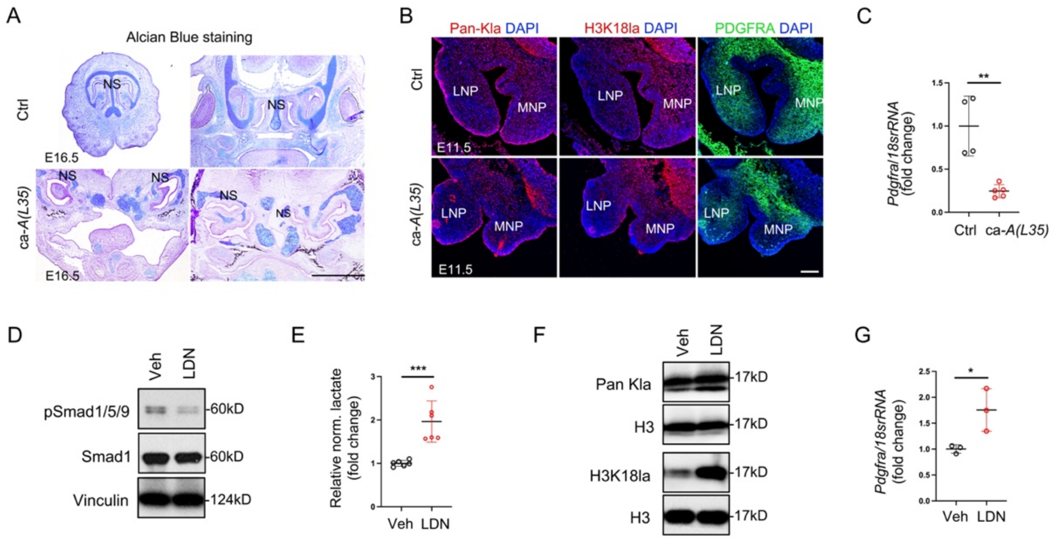

Figure S7 BMP-mediated regulation of *Pdgfra* histone lactylation is not restricted in CNCCs of ca-*Acvr1(A11)* mutants.

(**A**) Alcian blue staining of frontal sections of control and ca-*Acvr1(L35)* facial structures at E16.5 ($n = 4$). (**B**) Representative immunostaining of Pan Kla (red, left), H3K18la (red, middle), and PDGFRA (green, right) in control and ca-*Acvr1(L35)* NP tissues at E11.5 ($n = 5$). (**C**) Relative mRNA expression of *Pdgfra* in control and ca-*Acvr1(L35)* NP tissues at E11.5 ($n \geq 4$). (**D-G**) Representative immunoblots of pSmad1/5/9 and Smad1 (D, $n = 3$), relative normalized lactate levels (E, $n = 6$), representative immunoblots of Pan-Kla and H3K18la (F, $n = 3$, results shown are from blots run contemporaneously.), and relative *Pdgfra* mRNA expression (G, $n = 3$) in O9-1 cells treated with or without LDN193189. For all panels, data are presented as mean ± SD. ***$P < 0.001$, **$P < 0.01$, *$P < 0.05$, determined via unpaired 2-tailed Student's *t* test (C, E, G). Scale bars: 1 mm (A), 100 µm (B). NS, nasal septum, MNP, medial nasal processes, LNP, lateral nasal processes. See complete unedited blots in the supplemental material.

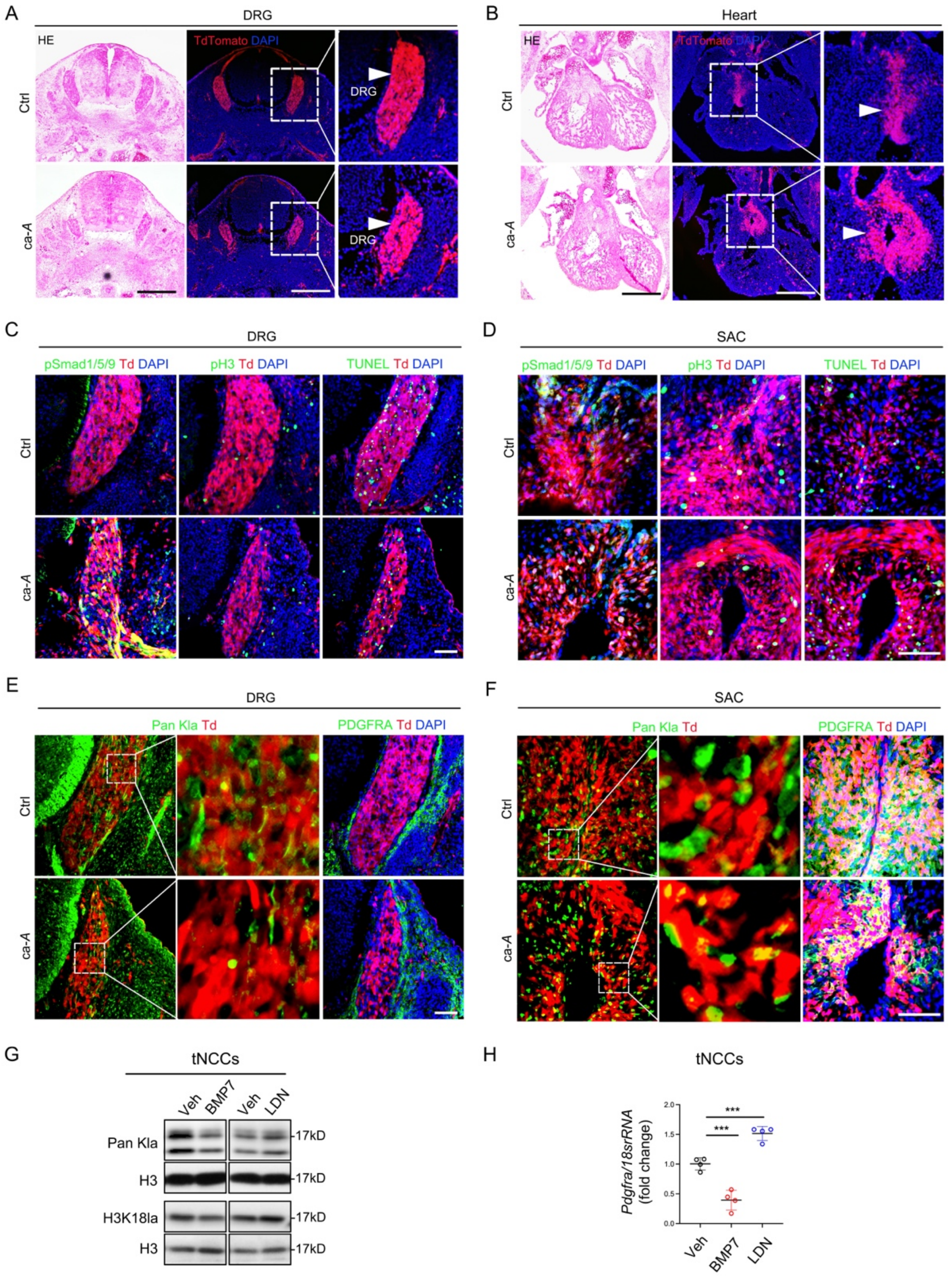

Figure S8 Augmented BMP signaling also caused developmental defects in trunk and cardiac NCC-derivatives accompanied with decreased histone lactylation and PDGFRA.

(**A, B**) HE (left) and DAPI (right) staining of transverse sections of DRG (A) and

heart tissues (B) of E12.5 embryos labeled with *R26R*^{tdTomato} (n = 5). (**C, D**) Representative immunostaining of pSmad1/5/9 (green, left), pH3 (green, middle), and TUNEL (green, right) of DRG (C) and heart tissue (D) in E12.5 embryos labeled with *R26R*^{TdTomato} (n = 5). (**E, F**) Representative immunostaining of Pan Kla (green, left and middle), and PDGFRA (green, right) of DRG (E) and heart tissue (F) in E12.5 embryos labeled with *R26R*^{TdTomato} (n = 5). (**G, H**) Representative immunoblots of Pan-Kla and H3K18la (G, results shown are from blots run contemporaneously), relative *Pdgfra* mRNA expression (H) in trunk NCCs (tNCCs) treated with BMP7 or LDN193189 (n = 4). For all panels, data are presented as mean ± SD. ***P < 0.001, determined via one-way ANOVA (H). Scale bars: 500 µm (A, B), 100 µm (C, D, E, F). DRG, dorsal root ganglion. White arrowheads indicate neural crest derived tissues (A, B). See complete unedited blots in the supplemental material.

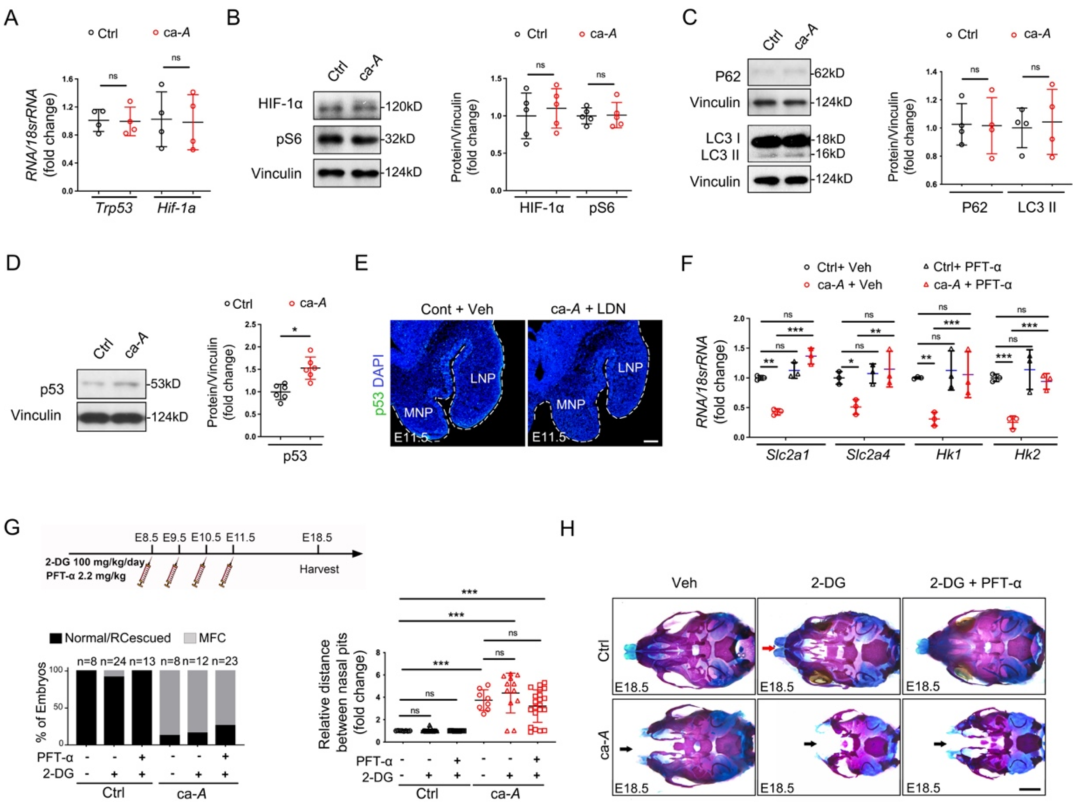

Figure S9 p53 functions as the upstream of glycolysis in the midline facial development.

(**A**) Relative mRNA expression of *Trp53* and *Hif-1a* in E10.5 NP tissues (n = 4). (**B-D**) Representative immunoblots and quantification of HIF-1α and pS6 (B, n = 5), LC3II and P62 (C, n = 4), and p53 (D, n = 6) in E10.5 NP tissues. Results shown are from blots run contemporaneously. (**E**) Representative immunostaining of p53 in NP tissues of embryos treated with or without LDN193189 (2 mg/kg/day, E8.5-E11.5, $n \geq$ 3). (**F**) Relative mRNA expression of *Slc2a1*, *Slc2a4*, *Hk1*, and *Hk2* in NP cells treated with or without PFT-α for 24 hr (n = 4). (**G, H**) Inhibitor injection schedule (G, upper), quantification of the embryo number and distance between nasal pits (G, lower), and whole-mount skeletal staining (H) of embryos treated with or without 2-DG (100 mg/kg/day, E8.5-E11.5) and PFT-α (2.2 mg/kg/day, E8.5-E11.5). $n \geq 8$. Black arrows indicate midline facial clefting. Red arrow indicates bifid nasal tip. For

all panels, data are presented as mean ± SD. ***$P < 0.001$, **$P < 0.01$, *$P < 0.05$, ns no significant difference, determined via unpaired 2-tailed Student's *t* test (A-D) or one-way ANOVA (F, G). Scale bars: 100 μm (E), 1 mm (H). MNP, medial nasal process, LNP, lateral nasal process. See complete unedited blots in the supplemental material.

ONLINE SUPPLEMENTAL TABLES

Supplementary Table S1. Genotyping PCR primers.

Supplementary Table S2. qRT-PCR primers.

Supplementary Table S3. ChIP-qPCR primers.

Supplementary Table. S4. Antibodies used in this study.

SUPPLEMENTAL EXPERIMENTAL PRECEDURE

Histology and immunostaining

For histology, staged embryos were dissected in ice-cold phosphate buffered saline (PBS), fixed for 2 hr in 4% paraformaldehyde (PFA) and embedded in optimum cutting temperature (O.C.T.) compound. Sections were cut at 10 μm and stained with hematoxylin and eosin (HE), safranin O and fast green, Alcian blue, or immunostained according to standard protocols. The following antibodies were used for immunostaining: anti-PDGFRα (1:300; R&D Systems), anti-pH3 (1:600, Millipore), anti-p53 (1:200, Cell Signaling Technology), anti-GLUT1 (1:100, Abcam), anti-HK1 (1:100, Cell Signaling Technology), anti-HK2 (1:100, Cell Signaling Technology), anti-pSmad1/5/9 (1:100, Cell Signaling Technology), anti-pERK (1:100, Cell Signaling Technology), anti-pJNK (1:100, Cell Signaling Technology), anti-pP38 (1:100, Cell Signaling Technology), anti-pTAK1 (1:100, Cell Signaling Technology), anti-Pan-Kla (1:200, PTM), anti-H3K18la (1:200, PTM), anti-SOX9 (1:200, Millipore), anti-OSX (1:300, Abcam), anti-Neurofilament (1:100, Santa Cruz). The levels of cell death were measured by employing the TUNEL method using an In Situ Cell Death Detection Kit (Roche). DNA strand breaks were labeled with fluorescein and visualized with FITC. Nuclei were stained with 4′,6-diamidino-2-phenylindole (DAPI).

Whole mount DAPI staining

Whole-mount DAPI staining was performed as previously described (1). Briefly, E8.5-E12.5 and E16.5 embryos were harvested, fixed for 2 hr in 4% PFA and then

incubated with 5 mg/ml DAPI in PBS overnight at 4 °C after washing three times with PBS. After three washes with PBS, the embryos were placed under a fully automatized Nikon Ti microscope, and pictures were taken every 3 µm in the z-axis to cover all embryo thicknesses. The maximum projection of all the z-axis images using Fiji software revealed the fine topological details of the embryos.

RNAscope in situ hybridization

To detect single mRNA molecules, RNAscope was performed on 15 µm sections of O.C.T. compound-embedded embryos at E11.5. The sections were dried for 10 minutes (min) at 40 °C and kept at −80 °C. In this study, one 3-plex positive control probe, Mm PPIB (ACD, 320881); one 3-plex negative control probe (ACD, 320871); and a probe against Mm-*Acvr1* (ACD, 312411) were used. In situ hybridization was performed according to the protocol of the RNAscope Multiplex Fluorescent Reagent Kit v2 (ACD, 320293). Briefly, sections were dried in an oven at 40 °C for 3-4 min prior to incubation in cold 4% PFA for 25 min. The slides were then dehydrated in 50%, 70% and 100% ethanol for 5 min each at room temperature (RT). After drying the slides for 5 min at RT, H_2O_2 was added, and the slides were incubated for 10 min at RT. For antigen accessibility, the slides were treated with Protease IV for 20 min at RT, incubated in boiling antigen retrieval solution (< 98 °C) for 15 min, washed in water twice, dehydrated in 100% ethanol and finally treated with Protease Plus for 40 min at 40 °C. After washing twice in PBS, the slides were incubated with C1 probes for 2 hr at 40 °C. The probes were detected with Opal 570 reagent (PerkinElmer, FP1488A). To quench autofluorescence due to the accumulation of lipofuscin or other protein aggregates, the slides were incubated with TrueBlack (Biotium, 23007) for 30 sec at RT. Before mounting the slices, DAPI was added to

label the nuclei. Images were acquired using a Nikon confocal microscope.

O9-1 cell culture

The O9-1 cells were kindly provided by Dr. Robert E. Maxson at the University of Southern California, Los Angeles, USA. The O9-1 cells were cultured under undifferentiating conditions by following a previously published protocol (2). The culture medium used consisted of Dulbecco's modified Eagle's medium (DMEM) supplemented with 15% fetal bovine serum (FBS), 0.1 mM MEM nonessential amino acids, 1 mM sodium pyruvate, 55 μM β-mercaptoethanol, 100 units/ml penicillin/streptomycin and 2 mM L-glutamine. Importantly, the medium was conditioned with growth-inhibited STO (mouse embryonic fibroblast cell line) feeder cells overnight, filtered (0.22 μm pore size) and further supplemented with 25 ng/ml basic fibroblast growth factor (R&D Systems, 233-FB) and 1000 U of leukemia inhibitory factor (Millipore, ESG1106). For the siRNA knockdown experiments, O9-1 cells were washed twice with PBS and transferred to culture medium without antibiotics 1 hr before transfection. Negative control siRNA (scrambled, 20 pmol per reaction; Thermo Fisher Scientific), *siLdha* (20 pmol per reaction; Thermo Fisher Scientific, s63417) was transfected into cells using Lipofectamine RNAiMAX (Thermo Fisher Scientific, 13778150) following the manufacturer's standard protocol. The inhibitors or ligands used to treat O9-1 cells were as follows: GNE-140 (3 nmol final concentration; Sigma-Aldrich, SML2580), LDN193189 (100 nmol final concentration), or BMP7 (100 ng/ml final concentration; R&D Systems, 354-BP-010). After transfection or inhibitor treatment, cells were harvested for cell proliferation assays, wound scratch assays, western blot analysis or qRT-PCR analysis at the indicated time points.

Isolation and culture of CNCCs and tNCCs

The isolation of CNCCs-derived cells from NP tissues (NP cells) was performed according to previously described methods with minor modifications (3). In brief, after isolating NP tissues from E11.5 embryos, the epithelia were removed using microdissection forceps after digestion with 10 U/ml dispase (Gibco, 1657335) for 10 min at 37 °C. Mesenchymal tissues were then dissociated into a single-cell suspension by digestion with the enzyme TrypLE (Thermo Fisher Scientific, 12605028) for 3 min at 37 °C and filtered to remove undigested tissues. Cells were cultured using DMEM/F12 medium (1:1) supplemented with 15% FBS and antibiotics. The isolation of tNCCs was performed according to previously described methods (4). Cells at passage 1-2 were used in experiments. For transfection, NP cells were transfected with plasmids overexpressing *Slc2a1* (Origene plasmid MR207871L4) and *Hk2* (Origene plasmid MR211170L4) using Lipofectamine 3000 reagent according to the manufacturer's instructions. Overexpression of GLUT1 or HK2 was confirmed by SDS PAGE followed by western blot analysis. Simultaneously, NP cells transfected with the empty pLenti-C-mGFP-P2A-Puro vector (Origene, PS100093V) were used as controls (*oe Neg*). NP cells or tNCCs were treated with inhibitors or stimulated with ligands at the indicated times and concentrations. The inhibitors or ligands used in this study were as follows: LDN193189 (100 nmol final concentration), sodium lactate (10 mmol final concentration; Sigma-Aldrich, 71718), PFT-α (10 μmol final concentration; Sigma-Aldrich, P4359), 2-DG (1 and 5 mmol final concentration; Sigma-Aldrich, D6134), sodium acetate (10 mmol final concentration; Sigma-Aldrich, S2889), PDGFA (100 ng/ml final concentration; R&D Systems, 221-AA-010), and BMP7 (100 ng/ml final concentration; R&D Systems, 354-BP-010).

Cell wound scratch assay

NP cells or O9-1 cells were seeded on Matrigel-coated cover slips in 12-well plates at a density of 1 × 10^5 cells per well. After reaching 70–80% confluency, the cells were starved for 24 hr in DMEM/F12 containing 0.5% FBS. In some experiments, serum-starved cells were pretreated with PDGFA (R&D Systems), 2-DG (Sigma), sodium L-lactate (Sigma), or sodium acetate (Sigma) or transfected with *Ldha siRNA*, *Slc2a1* or *Hk2* overexpression plasmids before scratching. After treatment with low-serum medium (DMEM/F12 containing 0.5% FBS) with 10 mg/ml mitomycin C for 2 hr to exclude the secondary effects of growth differences, the scratch was mechanically created using a sterile P200 pipette tip and washed twice with starvation medium to remove cell debris. The wound area was then photographed at marked positions (3 different fields per well). The cells were allowed to migrate for 24 hr at 37 °C before the same fields were recorded. All experiments were performed in triplicate. The scratch results were measured with ImageJ software (NIH, Bethesda, USA).

Cell proliferation assay

Cells were seeded at 5 × 10^3 cells per well in 96-well plates, and the cell number was determined using a standard crystal violet staining protocol (5).

In vivo inhibitor administration

LDN193189, PFT-α, 2-DG, and sodium-L-lactate were used for the treatment of animals. LDN193189 was dissolved in sterile endotoxin-free water (3). PFT-α was dissolved in 50% DMSO in PBS at 1.68 mg/ml and diluted ten times with PBS before injection (6). 2-DG and sodium-L-Lactate were dissolved in PBS. After optimizing the dosage and injection schedule, 2 mg of LDN193189, 2.2 mg of PFT-α, 200 mg of

2-DG, or 1 g of sodium L-lactate per kilogram body weight was used once per day. For single injection, 2 mg of LDN193189 was intraperitoneally injected into pregnant mice once at E9.5, E10.25, E10.75, E11.25 or E11.75, respectively. For continuous injection, each chemical was intraperitoneally injected into pregnant mice starting on days E8.5 to E11.5. Embryos at E18.5 were collected for whole-mount alizarin red and/or Alcian blue staining. Embryos at E11.5 and E15.5 were collected for western blot analysis, immunostaining and histological staining. With sodium lactate injection, pups at newborn (NB), postnatal day 1 (PN1), PN9 and PN42 were collected for gross morphology and body weight measurement. The litter size of females with sodium lactate injection was counted.

Whole-mount alizarin red and Alcian blue staining

After removing the skin and adipose tissue, embryo heads were fixed in 95% ethanol overnight, stained with Alcian blue solution (0.015% [w/v]), destained with 70 and 95% ethanol, and precleared with 2% potassium hydroxide solution. After being stained or not stained with alizarin red (0.015% [w/v]) for 2 hr, samples were destained in 20% glycerol/1% KOH solution and stored in 50% glycerol/47.5% ethanol solution before photographs were obtained under a stereomicroscope.

Lactate assay

Tissue or cellular lactate levels were measured using a lactate assay kit (Sigma-Aldrich, MAK064) in accordance with the manufacturer's protocol. Briefly, cells with different treatments or NP tissues from E11.5 embryos were washed with cold PBS and lysed in lactate lysis buffer. The lysate was collected and vortexed to ensure complete cell lysis. The samples were immediately frozen in liquid nitrogen and

stored at -80 °C or processed for assay. The values obtained from this assay were normalized to the total protein amount in each sample as measured using a Pierce BCA protein assay kit (Thermo Fisher).

Glucose uptake assay

For the glucose uptake assay, NP cells were cultured at 1×10^4 cells/well in a clear-bottom 96-well plate. Following an appropriate incubation, glucose uptake was measured using Glucose Uptake-Glo™ Assay (Promega, J1341) according to the manufacturer's instructions.

2-NBDG uptake assay

E11.5 control and ca-*Acvr1(A11)* mutant embryos were dissected and incubated in 500 µl of a 1 mM solution of the fluorescent glucose analog 2-NBDG for 1 hr at 37 °C. Following incubation, the embryo heads were fixed in 4 % PFA for 2 hr at RT and imaged using a fluorescence stereomicroscope.

Untargeted metabolomics

NP cells from control and ca-*Acvr1(A11)* embryos were cultured on 10 cm dishes. After reaching approximately 70% confluency, the cells were washed with 150 mM ammonium acetate (Sigma-Aldrich, A7330-100g) quickly (<30 seconds). The cells were then quenched by pouring liquid nitrogen directly onto the cell plate. Then, samples were processed using a single liquid-liquid extraction (SLLE) method with the addition of 1 mL of a chilled 1:1:1 (v/v/v) mix of acetonitrile (Supelco, AX0156-1), methanol (Supelco, MX0486-1), and acetone (Supelco, AX0156-1) containing a mixture of ^{13}C-labeled glycolysis internal standards at known concentrations for 10

min at 2-8 °C to precipitate the protein content. The samples were sonicated in chilled water for 10 min to increase the efficacy of precipitation. Centrifugation was performed at 12,000 RPM for 10 min at 4 °C to separate the precipitated proteins. An internal control sample was created by pooling equal volumes from each unknown sample to observe the continuity of the mass spectrometry output for final analysis. A volume of 200 µl from the resulting supernatant was brought to complete dryness using 100% nitrogen gas at the ambient temperature for 2 hr. The dried samples were then reconstituted with reconstitution solvent consisting of 20% methanol and an additional 2 internal standards (1:2 reconstitution solvent volume: dried sample volume). After a short burst of vortexing, the samples were centrifuged for clarification by pelleting any solute not removed during the SLLE process. The resulting volume was then analyzed by LC-MS for ion pairing reversed-phase (IPRP) analysis. The analysis was performed on an Agilent system consisting of an Infinity Lab II UPLC coupled with a 6545 QT mass spectrometer (Agilent Technologies, Santa Clara, CA) using a JetStream ESI source in negative mode. The metabolites were identified by matching the retention time and mass (+/- 10 ppm) to authentic standards. The peak areas were integrated using Profinder v8.00 (Agilent Technologies, Santa Clara, CA.) Nontargeted data analysis was performed using Agilent's MassHunter Find by Molecular Feature workflow (v7.0) with recursion using Agilent's Mass Profiler Pro (v8.0.) The dataset was processed using Binner1 to remove degenerate features, and the resulting features were searched against the Metabolomics Workbench Refmet database (https://www.metabolomicsworkbench.org/databases/refmet/index.php) to provide MSI2 Level III identifications or to an in-house library of authentic standards to provide MSI Level I identifications.

Seahorse assay for measuring ECARs or OCRs in live cells

Control and ca-*Acvr1(A11)* NP cells were isolated as described above, and the number of live cells was determined via a Trypan blue (Sigma-Aldrich, T8154) exclusion test. A total of 1×10^5 live cells per well were seeded in Matrigel-coated XFp cell plates (Agilent, 103022-100) and allowed to attach for 4 hr in culture medium. The cells were rinsed once with medium to discard nonattached cells.

To measure ECARs, cells were incubated for 1 hr in 180 μl of Seahorse XF complete DMEM supplemented with 2 mM glutamine (pH adjusted to 7.4). The microplate was incubated in a non-CO_2 37 °C incubator for 1 hr. Following incubation, the Seahorse assay was run in a Seahorse XFp analyzer according to the manufacturer's instructions. Inhibitors were used at the following concentrations: 1 mM oligomycin, 2 mM FCCP and 5 mM 2-DG (Agilent, 103015-100). ECAR measurement in NP cells subjected to BMP7 or PFT-α was performed in the same manner with the following exceptions: the cells were treated with BMP7 (100 ng/ml) for 4 hr or PFT-α (10 μM) for 24 hr and then dissociated, plated in XFp plates and allowed to attach for 24 hr.

To measure the OCRs, cells were incubated for 1 hr in nonbuffered XF Base DMEM minimal medium (Agilent, 103334) supplemented with 1 mM pyruvate (Thermo Fischer Scientific, 11360), 10 mM glucose (Sigma-Aldrich G7021) and 2 mM glutamine (Thermo Fischer Scientific, 25030) (pH adjusted to 7.4). The microplate was incubated in a non-CO_2 37 °C incubator for 1 hr. The OCR was measured on a Seahorse XFp Extra Flux Analyzer following the manufacturer's instructions. Mitochondrial inhibitors were used at the following concentrations: 1 mM oligomycin, 2 mM FCCP and 0.5 mM antimycin A/rotenone (Agilent, 103015-100).

After the ECAR and OCR measurements, the cells from each well were fixed with 4% PFA, stained with DAPI and quantified. The raw OCR and ECAR values were normalized for differences in cell number between individual wells. The basal ECAR, maximal glycolytic capacity, glycolytic reserve, basal OCR, maximal respiratory capacity, and ATP turnover were calculated as previously described (7).

Neural crest explant cultures

Neural crest explant cultures were performed according to previous reports (8). Briefly, neural tubes anterior to the first pharyngeal arch were dissected from E8.5 embryos. Following two brief washes in ice-cold PBS, the heads were sagittally split into two equal halves. The tissue was incubated in 0.5% trypsin/2.5% pancreatin in PBS for 5 min on ice and then in DMEM with 10% FBS for 10 min to stop the reaction. The head mesenchyme was carefully dissected and isolated with fine-tipped Dumont #5 tweezers, and the neural tube was transferred to fibronectin-coated cover slips in a 6-well plate. For NCC emigration assays, neural tubes were cultured in DMEM/F12 with 10% FBS for 6 hr. The explants were then treated with low-serum medium (DMEM/F12 containing 0.5% FBS) with 10 mg/ml mitomycin C for 2 hr. The culture medium was replaced with low-serum medium and maintained for 24 hr. The results were recorded at 8 hr and 24 hr.

RNA isolation, RNA-Seq and qRT-PCR

Total RNA was isolated using TRIzol reagent following the manufacturer's protocol. This method was employed to isolate RNA for qRT-PCR and RNA-seq analysis. For RNA-seq analysis, RNA was submitted to the Advanced Genomics Core, University

of Michigan. RNA-seq of E11.5 Control and ca-*Acvr1(A11)* NPs was performed using the Illumina HiSeq 2000 platform. Kyoto Encyclopedia of genes and genomes (KEGG) pathway enrichment analysis of differentially expressed genes was performed using DAVID. All differentially expressed genes were mapped to the KEGG database, and searched for significantly enriched KEGG pathways at $p < 0.05$ level. For qRT-PCR, first-strand cDNA was synthesized with 1 mg of denatured RNA using a SuperScript First-Strand Synthesis System (Thermo Fisher Scientific, 11904-018) with an ABI PRISM 7500 (Applied Biosystems) to measure the relative mRNA levels using a SYBR Green kit (Thermo Fisher Scientific, 4367659). Each reaction was performed in triplicate. The quantity of each experimental sample was first determined using a standard curve based on the cycle threshold (Ct) values and then expressed relative to the internal control. The data were normalized to *18S* rRNA levels using the $2^{-\Delta\Delta Ct}$ method. The DNA sequences of the primers used for qRT-PCR are summarized in Table S2.

ChIP assay

ChIP was performed on NP cells using a SimpleChIP® Enzymatic Chromatin IP Kit (Cell Signaling Technology, S9003) according to the manufacturer's instructions. Briefly, cells were harvested and resuspended in fresh culture medium, crosslinked with 1% formaldehyde for 10 min, and quenched with 0.125 M glycine for 5 min at RT. The harvested cells were washed twice with ice-cold PBS and then resuspended in nuclear preparation buffer. The nuclear pellet was harvested and resuspended in shearing buffer and sonicated on ice until the sheared DNA was approximately 200–1,000 bp in size. The samples were then centrifuged at 16,000 ×g for 10 min at 4 °C to remove debris, and the supernatants were diluted 1:1 in dilution buffer. The prepared

material was then used for protein/DNA immunoprecipitation. Antibodies directed against Pan-Kla, H3K18la, pSmad1/5/9, An antibody against total histone 3 (H3, positive control), or rabbit IgG (negative control) (Cell Signaling) were prebound to the assay wells, and immunoprecipitation reactions were carried out with chromatin extracts. Five percent of the chromatin extract was set aside as input. After immunoprecipitation, crosslink reversal was carried out, and the precipitated DNA was purified. The DNA was quantified by qRT–PCR analysis. All ChIP signals were normalized to the input, and the fold enrichment relative to IgG controls was compared. The primer sequences used to amplify the proximal promoter regions of the mouse genes are listed in Table S3.

Protein isolation and western blotting

For protein extraction, harvested NP tissues from each embryo or NP cells with different treatment were washed with PBS containing protease and phosphatase inhibitors (Thermo Fischer Scientific, 78420). The tissues were homogenized with Precellys Tissue Homogenizer (Bertin Instruments) in radioimmunoprecipitation assay buffer supplemented with deoxyribonuclease I and protease inhibitor cocktail. SDS–polyacrylamide gel electrophoresis (PAGE) and Western blotting were carried out according to standard protocols. Antigen detection was performed using the antibodies listed in Table S4. Bound primary antibodies were detected with horseradish peroxidase–conjugated species-specific secondary antibodies (Cell Signaling Technology) using a Super Signal Pico system (Thermo Fisher Scientific, 34079). The immunoreactive bands were quantified using ImageJ, and the mean ratios of the indicated protein from three independent experiments are shown at the bottom of the figures.

References

1. Sandell LL, Kurosaka H, and Trainor PA. Whole mount nuclear fluorescent imaging: convenient documentation of embryo morphology. *Genesis.* 2012;50(11):844-50.
2. Ishii M, Arias AC, Liu L, Chen YB, Bronner ME, and Maxson RE. A stable cranial neural crest cell line from mouse. *Stem Cells Dev.* 2012;21(17):3069-80.
3. Yang J, Kitami M, Pan H, Nakamura MT, Zhang H, Liu F, et al. Augmented BMP signaling commits cranial neural crest cells to a chondrogenic fate by suppressing autophagic beta-catenin degradation. *Sci Signal.* 2021;14(665).
4. Pfaltzgraff ER, Mundell NA, and Labosky PA. Isolation and culture of neural crest cells from embryonic murine neural tube. *J Vis Exp.* 2012(64):e4134.
5. Feoktistova M, Geserick P, and Leverkus M. Crystal Violet Assay for Determining Viability of Cultured Cells. *Cold Spring Harb Protoc.* 2016;2016(4):pdb prot087379.
6. Hayano S, Komatsu Y, Pan H, and Mishina Y. Augmented BMP signaling in the neural crest inhibits nasal cartilage morphogenesis by inducing p53-mediated apoptosis. *Development.* 2015;142(7):1357-67.
7. Zhang J, Nuebel E, Wisidagama DR, Setoguchi K, Hong JS, Van Horn CM, et al. Measuring energy metabolism in cultured cells, including human pluripotent stem cells and differentiated cells. *Nat Protoc.* 2012;7(6):1068-85.
8. He F, and Soriano P. A critical role for PDGFRalpha signaling in medial nasal process development. *PLoS Genet.* 2013;9(9):e1003851.
