# Supplementary material for: A BMP-controlled metabolic/epigenetic signaling cascade directs midfacial morphogenesis
Source: J Clin Invest. 2024 Mar 11;134(8):e165787. doi: 10.1172/JCI165787 (PMC11014657; doi:10.1172/JCI165787)

Full unedited blots for Figure 1H

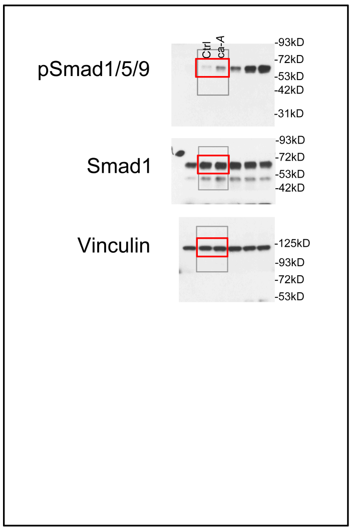

Full unedited blots for Figure 2F

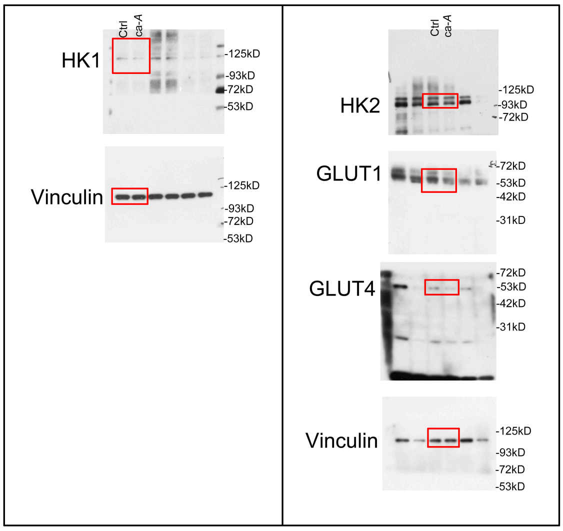

Full unedited blots for Figure 2G

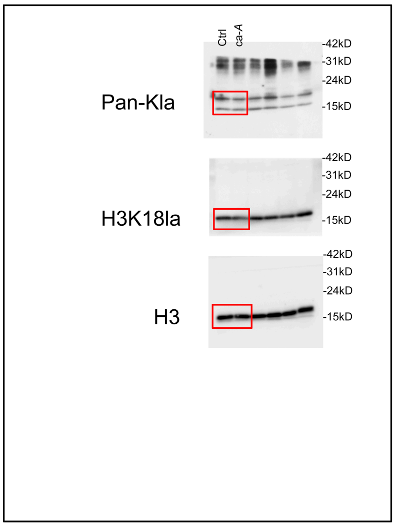

Full unedited blots for Figure 2I

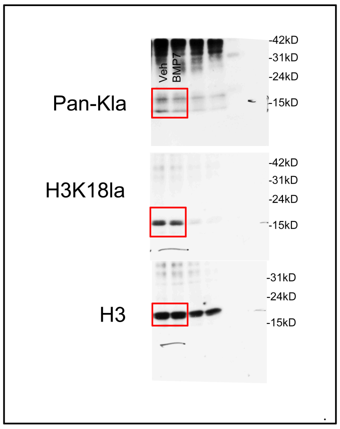

Full unedited blots for Figure 3A

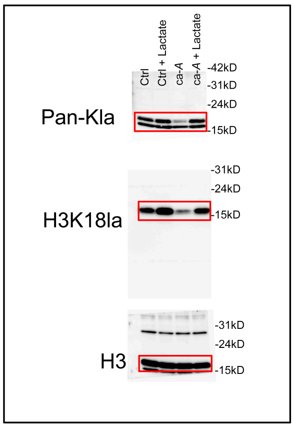

Full unedited blots for Figure 4B

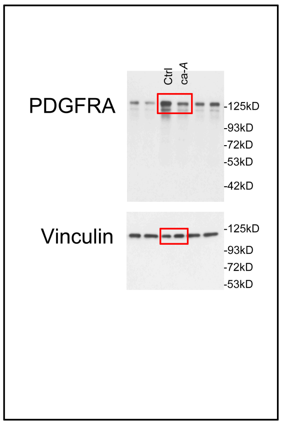

Full unedited blots for Figure 5F

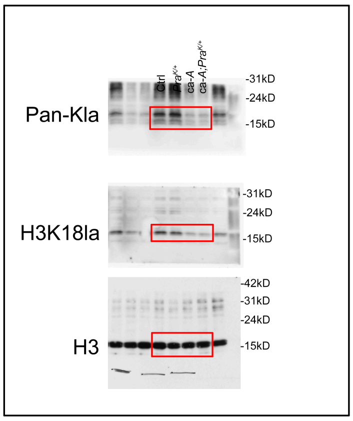

Full unedited blots for Figure S3D

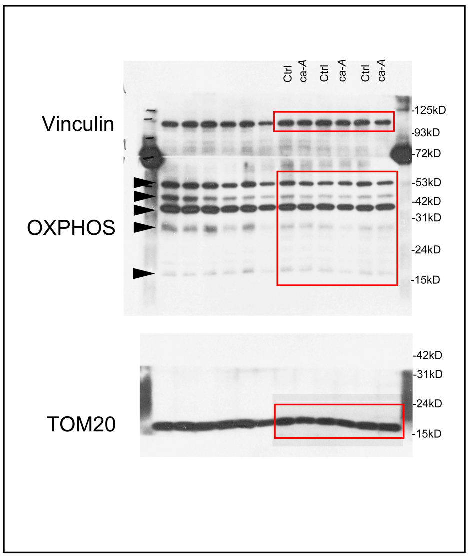

Full unedited blots for Figure S3L

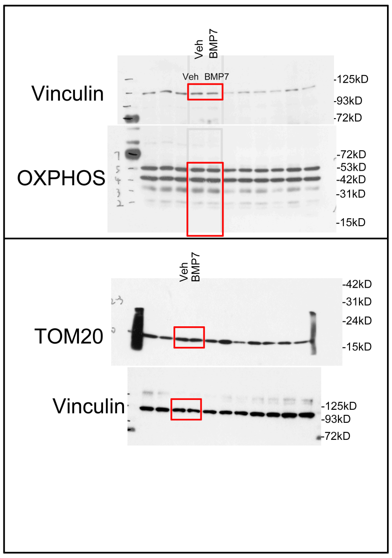

Full unedited blots for Figure S4A

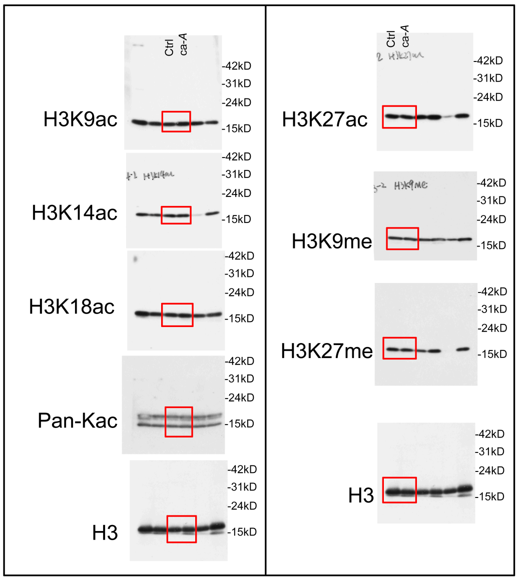

Full unedited blots for Figure S4E

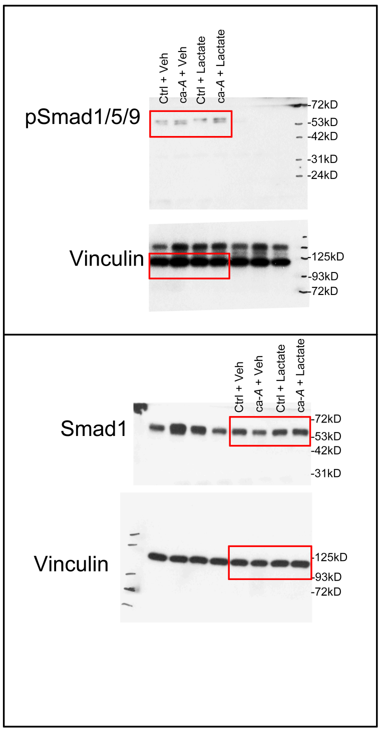

Full unedited blots for Figure S4F

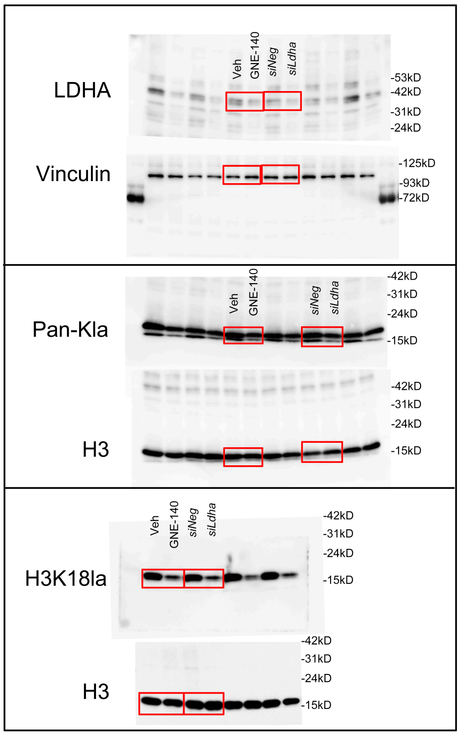

Full unedited blots for Figure S6A

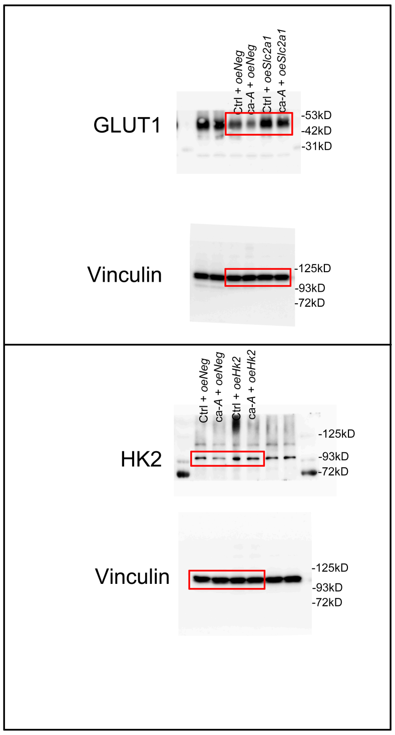

Full unedited blots for Figure S7D

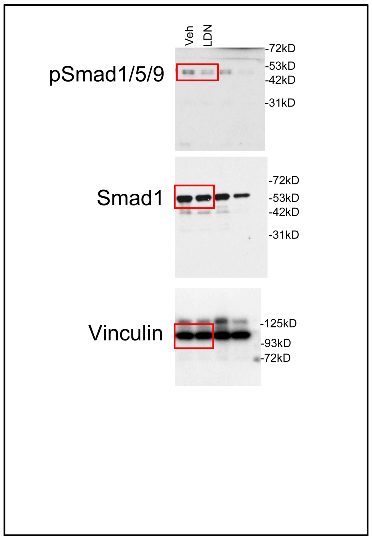

Full unedited blots for Figure S7F

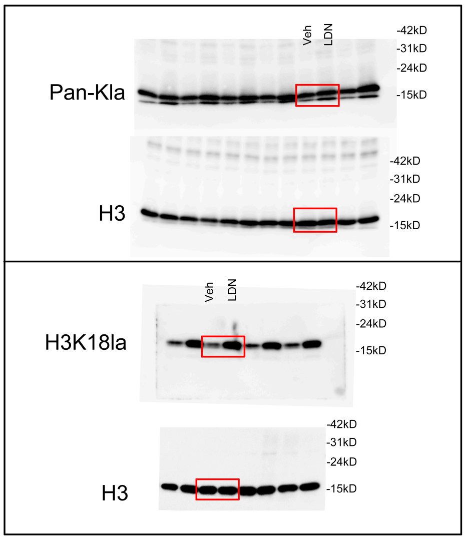

Full unedited blots for Figure S8G

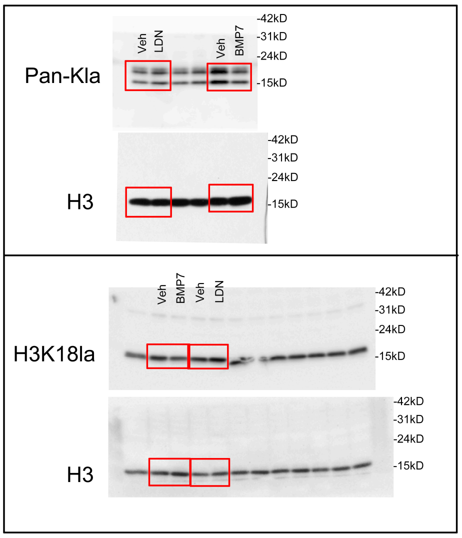

Full unedited blots for Figure S9B

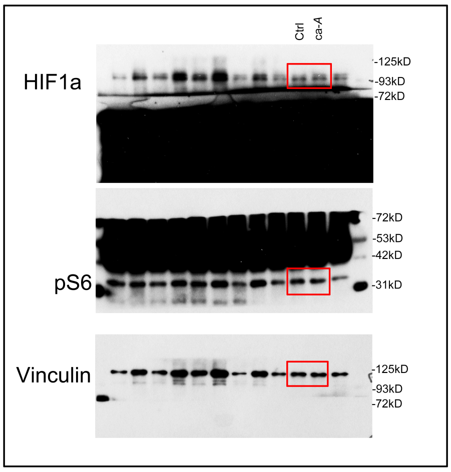

Full unedited blots for Figure S9C

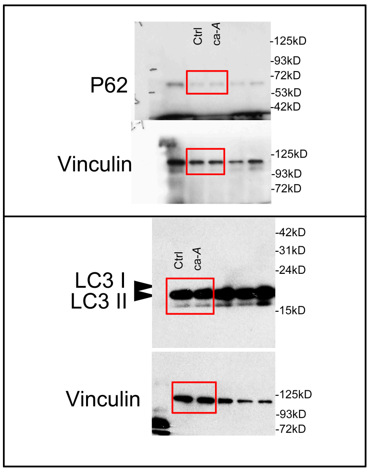

Full unedited blots for Figure S9D

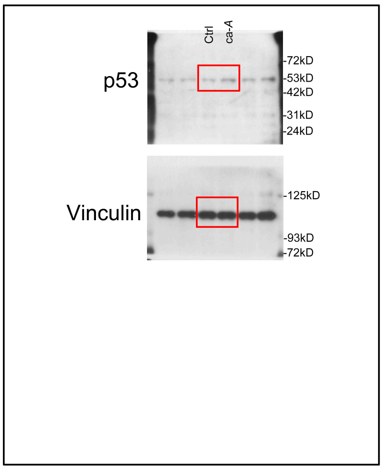

Supplement: Unedited blot and gel images [file jci-134-165787-s169.pdf]
